# Morphology dependent interaction between Co(ii)-tetraphenylporphyrin and the MgO(100) surface†

**DOI:** 10.1039/d0cp04859c

**Published:** 2021-01-04

**Authors:** Silviya Ninova, Osman Barış Malcıoğlu, Philipp Auburger, Matthias Franke, Ole Lytken, Hans-Peter Steinrück, Michel Bockstedte

**Affiliations:** Chemistry and Physics of Materials, Paris-Lodron University Salzburg Jakob-Haringer-Str. 2a A-5020 Salzburg Austria Michel.Bockstedte@jku.at; Theoretische Festkörperphysik, Friedrich-Alexander-Universität Erlangen-Nürnberg Staudtstr. 7B2 D-91058 Erlangen Germany; Physikalische Chemie II, Friedrich-Alexander-Universität Erlangen-Nürnberg Egerlandstr. 3 D-91058 Erlangen Germany; Institut für Theoretische Physik, Johannes-Kepler-Universität Linz Altenberger Str. 68 A-4040 Linz Austria

## Abstract

Porphyrins are key elements in organic–inorganic hybrid systems for a wide range of applications. Understanding their interaction with the substrate gives a handle on structural and electronic device properties. Here we investigate a single transition-metal porphyrin, namely Co(ii)-tetraphenylporphyrin (CoTPP), on the MgO(100) surface and the effect of multilayer film formation within hybrid density-functional theory and many-body perturbation theory. We focus on the relevant adsorption sites, simulate their photoemission spectra as a key fingerprint and compare with experiments on MgO(100) films on Ag(100). While we find only weak interaction between the cobalt centre and terrace sites on the MgO(100) surface, a strong interaction manifests itself with the low-coordinated sites. This leads to distinct features in both the valence and core-level regions of the electronic structure, as observed in the ultraviolet and X-ray photoemission spectra, corroborated by simulated spectra and calculated cobalt core-level shifts. Our work thus demonstrates the relevance of morphology-related low-coordinated sites and their properties in the adsorption of CoTPP on the MgO(100) surface.

## Abstract 2

The adsorption of Co-tetraphenylporphyrin at relevant low-coordinated sites on MgO(100) shows distinct features from terrace-site and multilayer films in the near-valence and corelevel regions of the electronic structure.

## Graphical abstract

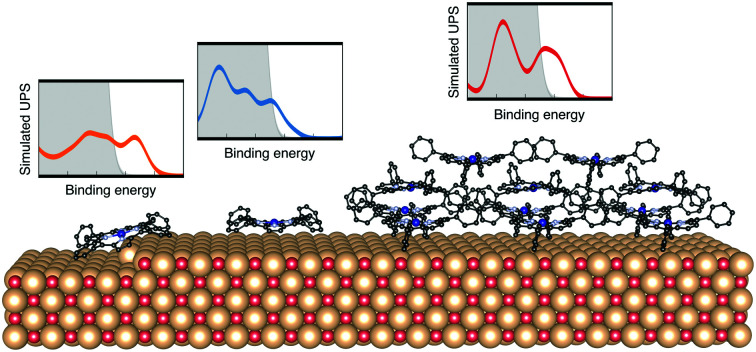

## Introduction

Porphyrin–substrate hybrid systems are the building blocks in a series of materials, such as the organic light-emitting diodes, chemical sensors, dye-sensitized solar cells and solar-energy conversions.^1–9^ Understanding therefore the way these molecules interact with the substrate upon adsorption holds the key to the prediction and improvement of the present-day devices.

Porphyrins are substituted porphine derivatives with a rather versatile chemical structure in terms of substituents and free-base/metalated macrocycle. Such a chemical flexibility is necessary to direct and control the interaction with the substrate on the molecular level only. The substituent bulkiness, for instance, can tune the adsorption strength in terms of distance from the surface^10^ and its chemical activity can influence the adsorbate orientation on the surface.^11,12^ The metal centres in the metaloporphyrins can also serve as an anchor to the surface, when chemical bonds are favoured over physisorption. Such interactions are known to lead to altered geometries and electronic properties,^13^ along with affecting the metal magnetic moment.^14,15^

The molecule–substrate interaction naturally depends on the surface morphology as well. The presence of atomically flat terraces,^16^ adatom layers,^17–19^ different adsorption sites^20,21^ or reconstructions^22–24^ strongly affects the adsorbate arrangement on the surface. Porphyrins tend to occupy first the low-coordinated sites, such as step-edges,^25,26^ where free-base porphyrins are known to undergo self-metalation.^6,27–29^ Physisorption, conversely, leads to a self-assembly into islands,^20^ governed by the van der Waals interactions between the porphyrins in the monolayer. The competition between molecule–molecule and molecule–substrate interactions is thus reflected in the monolayer structure.^30^

Such a variety of adsorption scenarios does not allow any *a priori* prediction about the resulting physical properties of the adsorbed molecules, which explains the great interest in those systems both at experimental and theoretical level.^6,31^ It is thus important to understand the extent to which an adsorbed porphyrin is affected by the contact with the surface in terms of geometry and electronic structure.

One member of the porphyrin family, namely the Co(ii)-tetraphenylporphyrin (CoTPP), has recently attracted considerable attention. Along with other porphyrin-derivatives,^32^ it was found to form a bound state with the underlying metal substrate, Ag(111)^10,14,15,33^ or Au(111),^34^ as well as on the Fe(001)-*p*(1 × 1)O surface.^19^ As a result the empty Co-3d_*z*^2^_ orbital fills up, quenching thus the CoTPP magnetic moment for the first adsorbed monolayer and forming a new state close to the Fermi level. This interaction with the surface can be controlled *via* the adsorption of NO on the metal centre in the porphyrin.^15,35–37^ The deposition of other metals,^38^ for instance, can conversely partially restore the magnetic moment on the CoTPP and thus make it a promising candidate for on-surface magnetochemistry.^39^

Interestingly, similarly strong surface interaction has been suggested between CoTPP and MgO(100).^40^ Indeed, ultraviolet photoelectron spectroscopy (UPS) experiments identify a peak close to the Fermi level for the monolayer deposition, which disappears for multilayers. Additionally, core-level states corresponding to the presence of both Co^0^ and Co^2+^ on the surface for low coverages are observed, suggesting a noticeable charge transfer from the surface to the molecule. This finding was assigned to the presence of two sites, distinct for their interaction with the adsorbate. Open questions remain regarding the adsorption strength of CoTPP on MgO(100), especially knowing that other tetraphenylporphyrins, MgTPP for instance,^16^ physisorb on this surface, and the extent to which such an interaction affects the mono- and multilayer formation and their electronic structure.

In this work, we investigate the interaction between a Co(ii)-tetraphenylporphyrin (CoTPP) and the MgO(100) surface within the density-functional (DFT) and higher levels of theory. First, we look into the possible adsorption sites on the atomically flat metal oxide surface and then explore the possibility of adsorption on other surface micro-structural features such as kinks and steps. Next, we suggest a scheme enabling the comparison between the mono-adsorbed CoTPP and the thin film in terms of their electronic structure. Based on simulated core-level shifts and UPS spectra we identify possible adsorption scenarios compatible with the reported experimental data.^40^ Finally, we look into the possibility of metal-exchange on the surface.

## Methods

### Computational details

Density-functional theory is a method widely used for the geometry and electronic-structure determination of adsorbates.^41^ Nevertheless, there are short-comings in the description of non-local exchange and correlation effects, such as dispersion forces, level ordering and gap renormalization of the HOMO–LUMO states upon adsorption on surfaces. This could lead to inaccurate adsorption geometries, energy-level alignments as well as incorrect charge transfer and requires higher levels of theory.

The many-body perturbation theory within the GW approximation^42^ has been used to accurately determine the level alignment in molecules, interfaces and molecular crystals.^43–47^ It captures important non-local correlation effects such as the image potential in adsorbate-substrate systems. The limiting factors of these calculations are their high computational cost and their strong dependency on the initial input in terms of Kohn–Sham orbitals along with the type of GW calculation.^48^ This is the reason for the recent development of several other level-alignment approaches,^49–54^ which are aimed to be competitive in accuracy and far less cumbersome.

In the present work we use different levels of theory for the different tasks. We performed the geometry relaxations using the semi-local functional PBE^55^ with Grimme's van der Waals corrections D3,^56^ which result in reliable geometries. The correct description of the electronic structure, however, requires the use of hybrid functionals for porphyrins and phthalocyanines. Only at this level of theory the HOMOs are macrocycle-derived states^32,57^ and not metal ones (see Fig. S1 in the ESI†). All reported projected densities of state (PDOS) are thus obtained with the HSE06 functional,^58^ where a fraction of exact exchange of 25% and the range-separation parameter *μ* = 0.2 Å^−1^ were used. Geometry optimization within the DFT+U method,^59^ which qualitatively corresponds to the hybrid results, does not deviate significantly from the PBE-one with maximum differences of 0.04 Å and 0.3° for the bond lengths and angles, respectively (see Section S3 in the ESI†), similarly to what was observed by other studies.^18^ Finally, we perform GW0 calculations on the gas-phase and crystal CoTPP as well as for the MgO(100) surface. With this approach, we obtain values for the ionization energies or electron affinities (where available) in very good agreement with the experimental ones. Corresponding results based on DFT using either local or hybrid functionals are far from such an excellent agreement (see Fig. S1 and Table S7 in the ESI† and below). GW0 calculations on the organic–inorganic system would be computationally too expensive, which is the reason why we adopted the DFT+Σ corrective scheme (for more details see Section “Simulated UPS spectra”).

We aim at describing the electronic structure of CoTPP adsorbed on MgO(100) as a mono- and multilayer. We thus work with two model systems for the two scenarios. The monolayer is described by a single or multiple adsorbed molecules, where the CoTPP is in direct contact with the MgO(100) surface, because we do not expect to have any intermolecular covalent interaction (see Section “On flat MgO(100)”). The multilayer, conversely, is approximated by the crystal CoTPP (see Section S4 in the ESI†), because the UPS spectra we compare to are not sensitive to the film-substrate interface. In order to compare these two systems with each other and with the experimental UPS spectra, we align their eigenvalues with respect to the common vacuum energy.

**Fig. 1:**
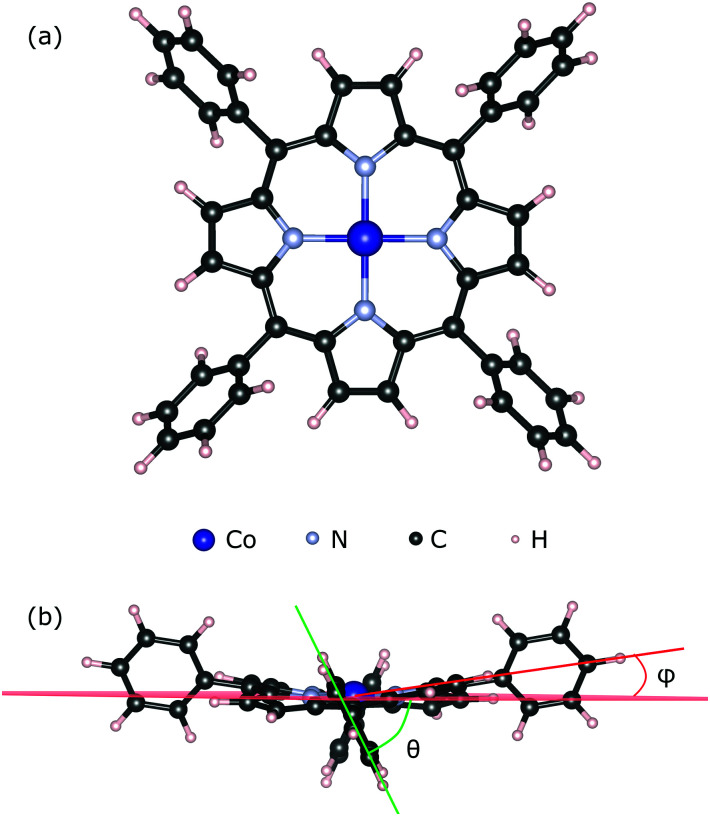
(a) Geometry structure of the Co(ii)-tetraphenylporphyrin; (b) phenyl angles with respect to the mean plane of the macrocycle: *θ* – twisting angle between the plane of the phenyl and that of the macrocycle, *ϕ* – out-of-plane distortion angle.

We used the CoTPP geometry as extracted from its crystal structure^60^ (see Fig. 1a). We relaxed its geometry in vacuum using several exchange–correlation functionals, all of which produce similar to the experimental structures (see Table S1 in the ESI†). The degree of molecular distortion was estimated with the help of the twisting angle between the plane of the phenyl and that of the macrocycle, *θ*, and the out-of-plane distortion angle, *ϕ* (see Fig. 1b). In the calculation of adsorption energies any thermal motion is neglected. Steric hindrances at the step-edge and kink sites can introduce correction terms due to the complex motion of the phenyls and the flexible macrocycle.

We implement the various surface micro-structures using suitable slab geometries. The flat terrace is represented by a 5 × 5 unit-cell^61^ slab in the surface plane, corresponding to an orthorhombic simulation cell with dimensions (21.194 × 21.194 × 27.000) Å^3^. The stepped and kink-containing surfaces were constructed so as to enable a reduction of the dipole created by the low-coordinated sites on each end of the slab. Their simulation cells are triclinic with the following dimensions: *a* = 30.808 Å, *b* = 27.169 Å, *c* = 20.990 Å, *α* = *β* = 90°, *γ* = 118.115° for the stepped surface and *a* = 30.808 Å, *b* = 27.942 Å, *c* = 23.410 Å, *α* = 92.362°, *β* = 92.499°, *γ* = 124.140° for the kink-surface. In addition, the charge transfer between the kink-atoms at the opposite sides of the slab was cancelled by the adsorption of a electron-donor (–H) or electron-withdrawing (–OH) group to the bottom oxygen and magnesium kink-ions, respectively. In all cases, the slabs are 5-atomic layers thick, where the bottommost one is kept fixed to the bulk positions.

The simulation cells were designed so as to accommodate one CoTPP molecule and thus avoid interactions between the periodic images in the surface plane. The distances to the periodic image on the terraced surface corresponds to ∼8.3 Å. The CoTPP on the stepped surface stays at ∼17.2 Å across the step and ∼7.8 Å along it away from its periodic repetition, while in the case of the kinked one the values are ∼14.7 Å across and ∼11.0 Å along the edge. Additionally, the vacuum region guaranteeing a surface calculation spans to more than 10 Å above the adsorbed CoTPP for all slabs.

All calculations were carried out with the Vienna Ab initio simulation package, VASP.^62,63^ The projector augmented plane-wave basis (PAW)^64,65^ is generated on the PBE functional and contains the following orbitals: Co-3d 4s; C-2s 2p; N-2s 2p; H-1s; Mg-3s; O-2s 2p. The energy cut-off was 600 eV and the augmentation one – 1200 eV. We carried out *Γ*-point calculations. All calculations are spin-polarized, due to the unpaired electron on the Co-3d_*z*^2^_ orbital. Electronic energies were converged better than 0.01 meV, whereas the geometry optimization one – at 5 meV Å^−1^ differences on the forces.

The GW0-approximation was used to obtain the level alignment in the gas and crystal phases, where the quasi-particle energies and one-electron orbitals were updated. LDA orbitals as a starting point were successfully used for H_2_TPP and MgTPP.^66^ In the description of CoTPP higher level of theory is required to correctly capture the orbital order in the valence region (see Fig. S1 in the ESI†). We thus chose the HSE06 Kohn–Sham orbitals as a starting point for our study. In the GW0 calculations for the molecule we used a supercell of (21 × 21 × 15) Å^3^ together with plane-wave and augmentation cut-offs of 421 and 905 eV, respectively. Additional virtual Kohn–Sham orbitals with an energy up to 80 eV above the HOMO level were included. Our setup has already been checked against the experimental results for other studies on MTPPs^66^ giving excellent agreement. These settings assure a relative convergence of the quasi-particle energies with respect to the HOMO better than 1.5 meV during the self-consistent GW0.

As far as the MgO(100) is concerned, both LDA and HSE06 starting points give similar results, comparable to the experimental band edges. This translates into an ionization potential (IP) of −7.0 eV (−7.1 eV) and a position of the unoccupied surface band edge at −0.7 eV (−0.4 eV) for G0W0@LDA (G0W0@HSE06) against the experiment with −7.0 and −0.8 eV.^67^ As the unoccupied states are not relevant here, we used the HSE06 values, so as to maintain a consistent starting point for all calculations. The G0W0 values were obtained using a slab model of MgO(001) with a 1 × 1 surface unit cell, seven layers, and a vacuum of 22.5 Å. The *k*-point sampling was based on a *Γ*-centered 8 × 8 × 1 *k*-point mesh and a PAW basis with an energy cut-off of 600 eV was employed. The virtual orbitals cover an energy range of 145 eV. The valence, surface and conduction band-edge positions are converged better than 2 meV.

All geometry structures were visualized with VESTA.^68^

### Experimental details

In the experiments, CoTPP was adsorbed on thin (100)-oriented MgO films grown on an Ag(100) substrate. The MgO film had a thickness of 10 monolayers, which sufficiently decouples the surface from the metal substrate. X-ray (XPS) and ultraviolet (UPS) photoemission spectra of the adsorbates were measured for submonolayer coverages and multilayer films. The procedures for the surface preparation and CoTPP deposition are reported in Franke *et al.*,^40^ along with the parameters to obtain the indicated XPS and UPS spectra.

## Results and discussion

### On flat MgO(100)

The atomically flat MgO(100) consists of oxygen and magnesium ions arranged in a chequerboard pattern typical for rocksalt surfaces. Plausible adsorption sites comprise bridge and the on-top ones on either of the two ions. Electrostatic interaction arises between the partial charges on the pyrrole-like groups and the Co(ii)-core and the surface ions. Chemical bonding, if any, between the surface and the adsorbate is expected to be strongest with the metal core and the oxygen.^17,40^ The lack of linking groups at the phenyl rings of CoTPP prevents any other chemical bond formation with the substrate. From here on we use the site nomenclature to the Co-position with respect to the surface.

**Fig. 2:**
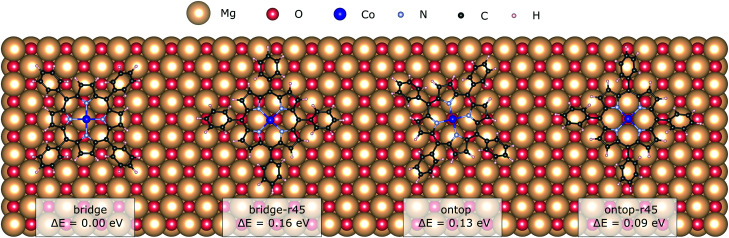
The two adsorption sites, bridge and ontop, with the different adsorption configurations as obtained after a geometry relaxation.

In this section we explore the adsorption with starting positions for the relaxation on both bridge and ontop oxygen sites and the effect of the resulting adsorption sites on the CoTPP. In addition, we look into the CoTPP orientation with respect to the Mg/O grid by namely trying rotations of 45° (*e.g.* bridge-r45, ontop-r45) (see Fig. 2).

We establish only small energy differences between these four CoTPP adsorption models on the flat MgO(100) after their geometry relaxation (see Table 1). This suggests a rather mobile adsorbate not anchored to a specific adsorption site. Indeed, the CoTPP remains relatively far from the substrate at ∼3.3 Å. The formation of a shorter chemical bond between the cobalt and surface oxygen, as encountered on other surfaces (see Table S3 in the ESI†), would thus inevitably require the deformation of the macrocycle. Constraining the Co–O bond distance to 1.9 Å leads to a geometry distortion of 2.00 eV. Overall, the enforced interaction does not compensate for the deformation energy, resulting in a decreased adsorption energy of 1.34 eV. The scenario remains thus implausible to occur.

**Table 1:** Geometry parameters for the CoTPP optimized on the MgO(100). The distance between the cobalt and the MgO surface is calculated from the averaged positions along *z* of both Mg and O. All energies are in eV, distances in Å and angles in °

Site	Ads. en.	*d*_Co–MgO_	*d*_Co–O_
Bridge	3.43	3.50	—
Bridge-r45	3.26	3.42	—
Ontop	3.30	3.21	3.11
Ontop-r45	3.33	3.22	3.12
Step-edge	4.34	—	2.17
Kink-O	4.42	—	1.90
Kink-Mg	4.82	—	2.97^a^

a *d*_Mg–N_ = 2.46 Å.

**Fig. 3:**
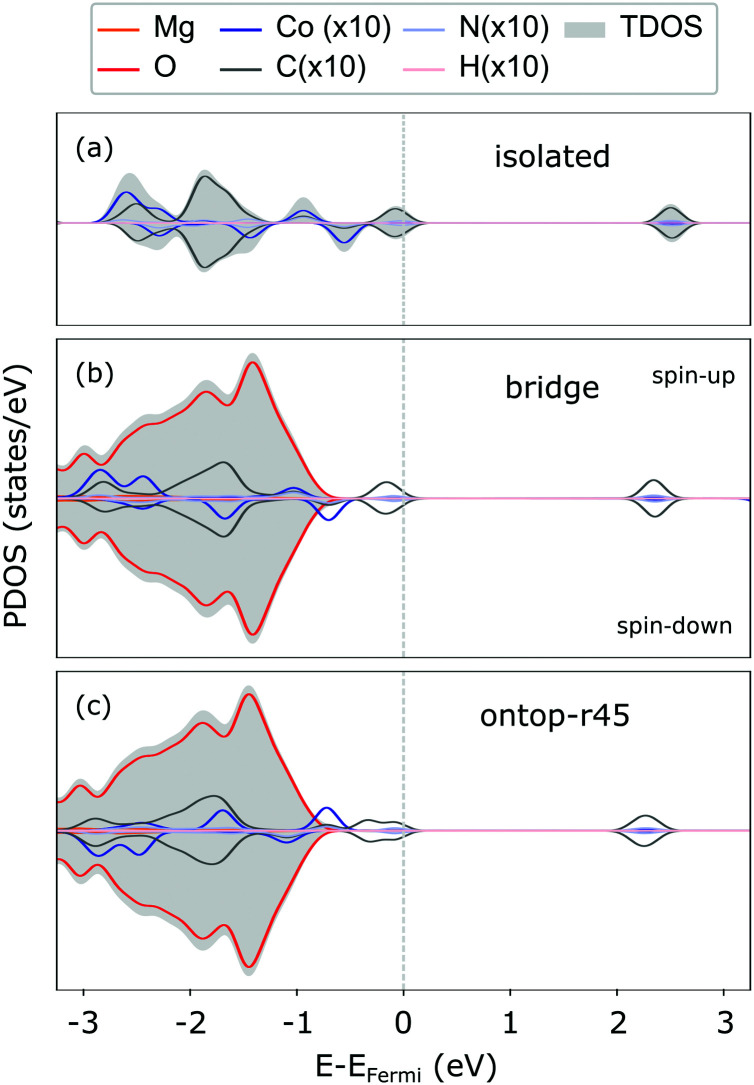
PDOS for the CoTPP in gas phase (a), at bridge (b) and ontop (c) sites on the flat MgO(100) as calculated using the HSE06 functional. The contributions coming from the adsorbant have been enhanced, so as to increase their visibility. The PDOS is spin-resolved with the spin components as indicated in panel (b). The Gaussian width 0.1 eV was used for the PDOS convolution.

The weak interaction with MgO is also reflected in the CoTPP electronic structure obtained with the HSE06 functional. It remains indeed almost unaltered with respect to the gas phase (see Fig. 3), suggesting little to no hybridization with the surface states. The valence region is dominated by molecular states – macrocycle-HOMOs and lower-lying Co-states – whereas the MgO states appear at −1 eV below the HOMO of the molecule. As opposed to the CoTPP adsorbed on Ag(111) for instance,^15^ our simulations on the electronic structure do not give any indications of a Co-state appearing as a result of a strong interaction with the surface (see Fig. 3). This weak surface–adsorbate interaction leads to an almost indistinguishable adsorption at the two sites.

The monolayer formation is often not only affected by a possible chemical bond of the central metal ion with the surface,^24^ but also by the surface structure, the phenyl substituents^69^ and molecular interactions governed by the metal core.^18^ The tetraphenylporphyrins tend to form a close-packed molecular layer as a result of van der Waals interactions between the phenyl rings.^20,33^ Interestingly, it was recently found that the monolayer formation enhances the interaction with the underlying oxide substrate.^21^ In this regard, we investigated this aspect for our model system (see Section S2.2 in the ESI† for more details on the simulation model). As opposed to the case reported in ref. 21, the CoTPP monolayer on the flat MgO(100) is farther away from the surface, yet in agreement with results for the CoTPP adsorption at Fe(100)-*p*(1 × 1)O^18,19^ (see Table S3 in the ESI†). The monolayer at MgO(100) is by only 0.08 eV more strongly bound to the surface than the isolated molecule. Its formation is driven by the favourable intermolecular van der Waals interactions, rather than by the substrate–adsorbate interaction. In our case, this leads to 0.58 eV gain in energy due to the adsorbate–adsorbate interaction. Overall, we find that a CoTPP monolayer interacts in a similar manner with the MgO as the isolated molecular adsorbate. This also justifies our approach.

### On stepped MgO(100)

**Fig. 4:**
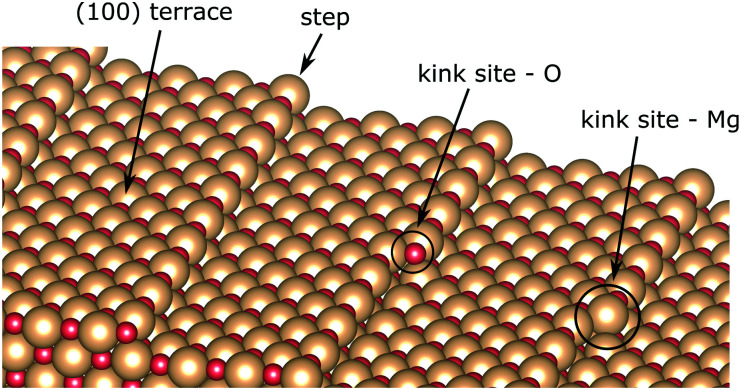
MgO(100) surface model with steps and distinct kink sites.

The MgO surface is characterized, though, not only by terraces in the (100) plane, but it is also dotted with micro-structural features upon its growth on the Ag(100) substrate (see Fig. 4).^67,70–72^ Several methods have been developed to improve the quality of the MgO thin films, such as annealing after Mg evaporation in O-rich atmosphere,^40,70,73^ increased growth temperature^29,74^ or cooling after growth.^75^ Such protocols efficiently reduce the abundance of surface defects but cannot entirely remove them. On the other hand, the low-coordinated sites, such as steps or kinks, render the surface chemically more active. The free-base porphyrin is reported to undergo metalation at those adsorption sites.^26,27,29^ In this regard, we explore the CoTPP adsorption on MgO(100) slabs containing steps and kinks. We exclude from the discussion surface point defects, considering their expected low concentration, generally below 0.1% per monolayer,^76^ and high formation energy in the range of several electronvolts.^77,78^

The steps in MgO(100) are mainly monoatomic^70^ and could be both non-polar and polar. The former constitute of both ions, whereas the latter are grown in the [011] direction, forming thus either magnesium- or oxygen-only steps. In the present study, we use a slab model with non-polar steps, as these are the most abundant ones.^72^ The terrace size was chosen, so that the accommodated CoTPP is not affected by its periodic image, corresponding thus to the stepped surface plane (017) with a terrace ∼14.6 Å wide. Such a step is determined to account for an increase in the surface energy of 0.23 J m^−2^ with respect to the atomically flat one.^79^

**Fig. 5:**
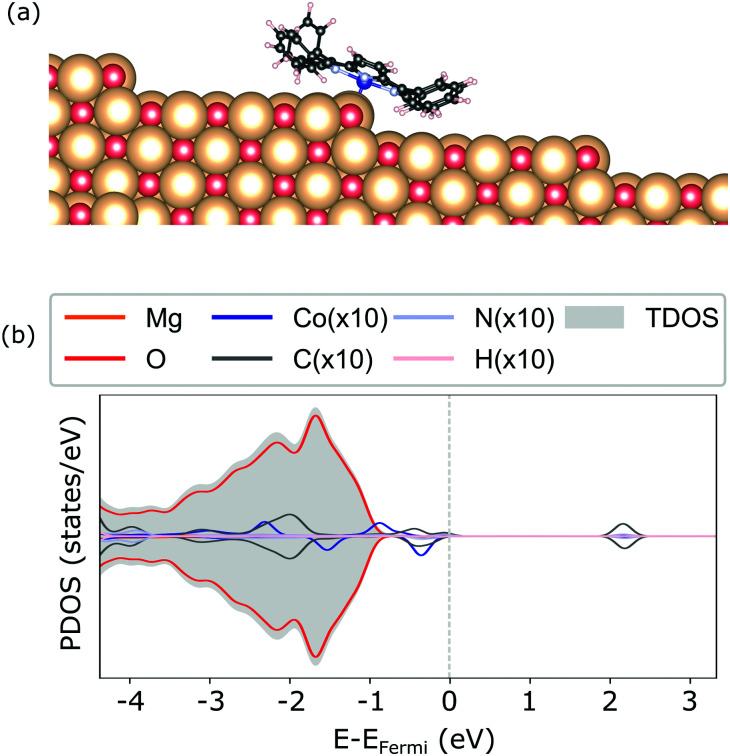
Geometry (a) and electronic structure obtained with the HSE06 (b) of the CoTPP adsorbed on the step-edge of MgO(100).

For the adsorption of CoTPP at the step-edge, we started with an initial configuration straddling the two terraces with the metal ion on top of the oxygen. The lattice site at the step-edge is less coordinated than the terrace one. It is indeed more reactive and it forms a chemical bond with the Co-ion, shorter with respect to the terrace by ∼1 Å. The overall CoTPP adsorption is stronger by about 1 eV (see Table 1). In addition, several changes are observed in the electronic structure. The HOMOs are not strictly separated into macrocycle and metal ones, as is the case for the gas-phase CoTPP (see Fig. 5). Indeed, a new state appears close to the Fermi level as a result of the interaction between the Co-3d_*z*^2^_ with an oxygen 2p, in agreement with the formation of a bond. This is accompanied by a small increase in the Bader charge^80^ transferred to the CoTPP at the step-edge adsorption site as compared to the terrace one, 0.37 *e vs.* 0.24 *e*, respectively.

**Fig. 6:**
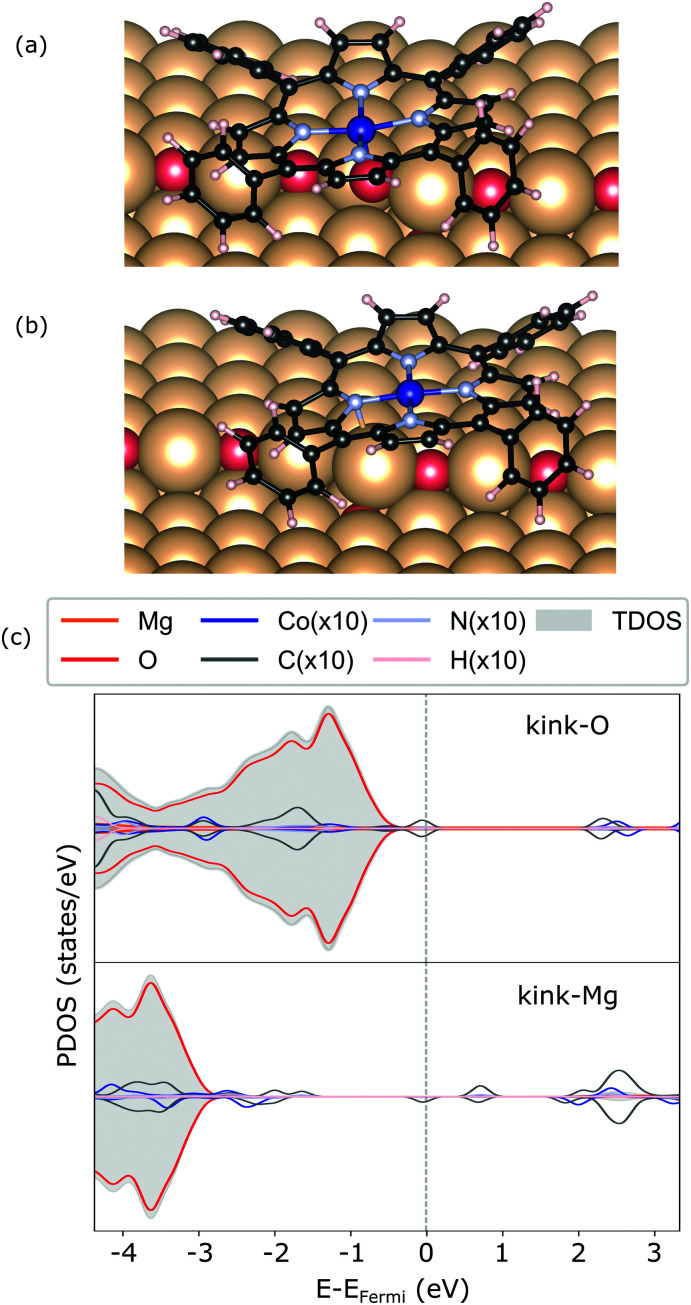
Geometry of CoTPP on kink-O (a) and kink-Mg (b), along with their electronic structures calculated with HSE06 in (c).

In addition, two kink-sites can be distinguished with a three-coordinated oxygen (kink-O) or magnesium (kink-Mg) ion (see Fig. 4). The CoTPP adsorption on the kink-O is similar to the one on the step-edge, where a Co–O bond is formed with a length of 1.90 Å (see Table 1 and Fig. 6a). This is accompanied by a displacement of the oxygen ion and its underlying magnesium slightly out of its edge plane. The adsorption of the metalotetraphenylporphyrin on the kink-Mg results in a shorter bond formation between the kink site and one of the macrocycle nitrogen – 2.46 Å and compared with the 2.97 Å Co–O in the same structure (see Fig. 6b). Interestingly, this adsorbate–substrate system has the highest adsorption energy of all cases studied here (see Table 1), whereas the adsorption energy on kink-O lies 0.40 eV lower.

In terms of the electronic structure, pronounced charge transfer is observed on both kink sites, as opposed to the flat or stepped surfaces. Indeed, the kink-O is strongly under-coordinated and the adsorption of CoTPP leads to the loss of 0.19 *e* (Bader charge), with the cobalt losing 0.21 *e*, as compared to the same molecule adsorbed on the terrace. This results in quenching of the metal magnetic moment (see Fig. 6c). In comparison, the Co-atom at the kink-Mg is less affected. The unpaired electron of the neutral kink-Mg site appears to charge the macrocycle LUMO state (see Fig. 6c). Indeed, the charge on the CoTPP is −1.20 *e*, as opposed to the 0.19 *e* on the kink-O or −0.24 *e* at the bridge site.

All things considered, we conclude that the adsorption of CoTPP with MgO(100) includes terrace and low coordinated sites as provided by the surface morphology, similarly to other porphyrins.^22,25,26,81^ STM images of MgO films grown on Ag(100) indeed show^82^ that step edges with defects and kink sites are largely abundant on these surfaces.

### Core-level shifts

**Fig. 7:**
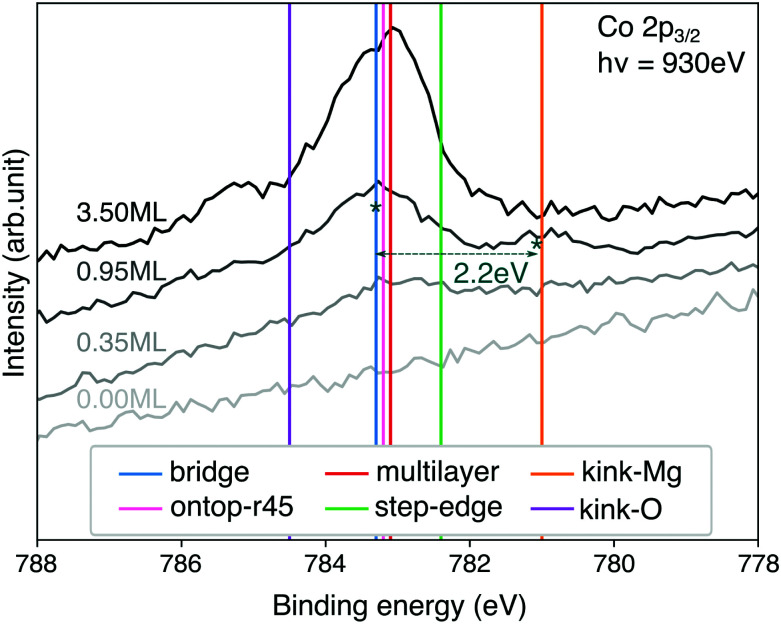
Comparison of XPS spectra at different CoTPP coverages with theoretical binding energies for Co 2p. The theoretical value for the bridge position is aligned to the experimental peak for 0.95 ML at 783.3 eV. The shift of 3.8 eV is applied consistently to the other calculated structures. The absolute non-shifted values are reported in the ESI,† Table S8.

The CoTPP monolayer exhibits two pronounced Co 2p_3/2_ core-level peaks at 783.3 and 781.1 eV, as opposed to the multilayer, where only one is observed at 783.1 eV (see Fig. 7).^40^ In this regard, we determined these core electron binding energies, so as to estimate their dependence on the different adsorption sites. The calculations are within the final-state approximation, the implementation of which in VASP includes the excitation of a single core electron to the lowest empty state and introduction of an additional charge in the ionic PAW potential.^83^ The valence electrons are relaxed, thus accounting for the screening effects, whereas the core ones are kept fixed. Our calculations are within the Janak–Slater transition state approximation, where only half an electron is excited.^84,85^ This method is known to give values closer to the experimental ones for 1s shifts in gas-phase molecules for instance.^86^

We chose to align the core-level energies with respect to the common and prominent MgO O-2p peak in the valence band. In the case of the CoTPP multilayer, which we approximate to the CoTPP crystal, we employed a commonly used vacuum-level alignment with respect to the bare MgO surface (see Section S4 in the ESI†). Such a choice can be justified because working with different slab models makes the alignment of the core-level energies with respect to the Fermi level challenging. Using the vacuum level^87^ could be equally demanding, considering the presence of a small dipole between the two sides of the slab.

Similarly to our conclusions on the electronic structure, we do not observe any differences in the Co core-levels of the CoTPP adsorbed on the flat MgO, namely bridge and top (see Fig. 7 and Table S8 in the ESI†). The thin film formation likewise only slightly affects the CoTPP core-level shifts. The low-coordinated sites, on the other hand, inflict more significant changes, as a result of the bonding. It is important to point out that we exclude any charge transfer coming from the Ag(100) substrate to the CoTPP as a result of the significant thickness of the oxide film. In the case of kink-Mg we obtain a lower value of −2.3 eV, which is close to the experimentally observed −2.2 eV^40^ (see Fig. 7), whereas the binding energy is higher by 1.2 eV on the kink-O.

The experimental XPS spectrum exhibits two features at a coverage of 0.95 ML (see Fig. 7). The main feature at 783.3 eV is at the position of the porphyrin multilayers and agrees with the calculated binding-energy position of molecules adsorbed on terrace sites. The second feature at 781.1 eV, with a contribution of roughly 20%, is in almost perfect agreement with the binding-energy position calculated for kink-Mg sites. Every fifth molecule at kink sites may seem high, but STM images of MgO(100) thin films on Ag(100)^74,82^ show a high density of MgO islands with lateral dimensions between 4–13 nm. Tetraphenylporphyrin molecules usually adsorb in square arrangements with a unit cell size of 1.4–1.5 nm, which means 9–64 molecules would fit on each island. Only 3–13 kink-Mg sites per island, which seems reasonable, would therefore be needed for the behaviour we observe.

Given the energy difference of 1.5 eV we calculate between terrace and kink-Mg sites, we would expect the molecules to preferentially decorate the kink-Mg sites first. However, experimentally we observe no preferential decoration of the kink-Mg sites at low coverage (see Fig. 7). One explanation for this could be entropic effects. The molecules on the terraces are likely highly mobile, behaving like a two-dimensional gas at lower coverages, whereas molecules at kink sites are likely fixed in position. However, even in the extreme assumption of completely freely translating and rotating molecules on the terraces, entropy can only account for 0.4 eV at 300 K. Even considering the uncertainty of the calculated adsorption energies, entropy is thus not enough to explain the discrepancy between the calculated energies and the observed behavior.

We believe the presence of hydroxyl groups at the MgO steps in the experimental measurements to be a plausible explanation. Hydroxyl groups are not stable on the terraces of MgO(100) at 300 K, but they are readily created at steps through the adsorption and dissociation of residual water from the background of the vacuum chamber.^79,88^ It would not be unreasonable for hydroxyl groups to partially passivate the steps and weaken the adsorption at kink-Mg sites to where entropy makes terrace and kink-Mg adsorption equally favourable. A full theoretical treatment of adsorption of CoTPP on the hydroxylated surface is presently too complex and beyond the scope of the present work.

### Simulated UPS spectra

We look next at how the low- and high-coordinated sites affect the electronic structure, differences of which are already observed at hybrid-functional level of theory. These calculations, however, do not account for the long-range correlation effects in adsorbate–substrate systems^89^ or in molecular crystals, which in both cases result in a gap renormalization. We thus need a method to describe both systems to the required accuracy, so as to be able to make a direct comparison between the CoTPP in a mono- or in multilayers.

The procedure of adjusting molecular state energies upon adsorption using model and correction terms from higher-level methods is not uncommon. It requires first the correct description of the molecule and substrate as isolated systems. This can be achieved using either GW calculations, OT-RSH functionals^52,90,91^ or even standard functionals where additional corrections are applied.^50^ Indeed, the molecular states are adjusted to match either the experimental ionization potentials/electron affinities^21^ or those calculated using Δ*SCF*.^92^ The scissor effect could be added as a whole to the occupied/unoccupied states^50^ or applied individually to each Kohn–Sham level.^44^

Once adsorbed on the surface, the effect of the surface polarization needs to be included, so as to account for the molecular band-gap narrowing by image-potential effects. The molecular gap renormalization strongly depends on the surface and the contribution of the chemical bonding. In the case of metals, it is a result of the surface polarization energy and scales with the density of states at the Fermi energy,^45^ while for semiconductors it is governed by the band gap.^93^ The amount of screening can be estimated with the help of the classical image-potential model,^94^ through a fit of the exchange–correlation potential to the classical image plane^52^ or even the electronic potential change as a result of the presence of the positively/negatively charged molecule^95^*via* Wannier functions^53^ or constrained DFT.^51,96^

**Fig. 8:**
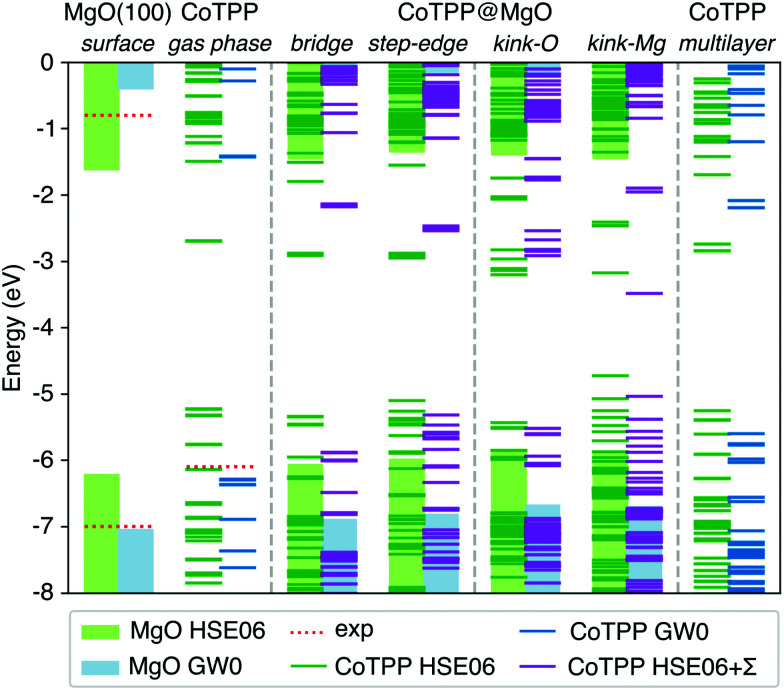
The energy level alignment for the MgO surface and CoTPP in gas phase, as adsorbed or in the multilayer, is presented as obtained at different levels of theory. The bars represent the position of the substrate valence and surface bands and the lines the strongly-localized molecular states. The vacuum energy has been chosen as a reference. The GW0 results for the MgO surface and the CoTPP in gas phase offer better agreement with the experimental ionisation potentials as opposed to the HSE06 (*cf.* text). The numerical values are in Table S7 in the ESI.†

In our study, we determined the level alignment for the isolated systems (MgO surface, CoTPP in gas phase and in crystal) within the GW0 approximation. At this level of theory we observe an excellent agreement with the experimental ionisation potentials (see Fig. 8) and the crystal UPS spectra over a wide energy range (see Fig. S6a in the ESI†). In comparison, the IP values obtained with the hybrid HSE06 functional deviate by as much as 0.8 eV, which invalidates its suitability for the energy level alignment.

The adsorbed system has been corrected following the DFT+Σ scheme.^49,50,52^ Given the reliability of the GW0 results, we expect that this approximate scheme to correctly predict the energy level alignment. Both surface and molecular states are shifted, so as to match their corresponding energy difference between the hybrid and the GW0 calculations. In addition, we adopt a point-charge model, where the polarization energy is given by^93^1
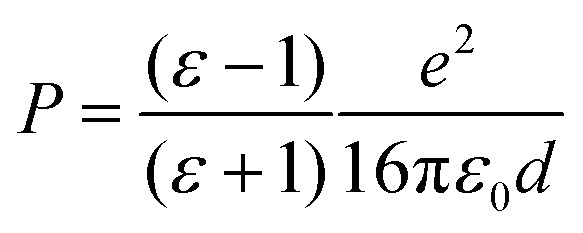
where *ε* is the dielectric constant of MgO, the electronic part of which is estimated to be 3.15; *ε*_0_ is the vacuum permittivity, *e* is one electron charge, *d* is the distance to the surface.

While there is a difference in the bonding between the adsorption on the terrace and low-coordinated sites, we assume in a first approximation that the point-charge model is still valid as a result of the localized character of the molecular HOMOs. The space separation between the CoTPP and MgO is estimated in terms of the Co–MgO distance for simplicity, considering that the metal ions lies within the centre of mass of the macrocycle. This results in polarization energies of 0.53 eV (3.50 Å), 0.86 eV (2.17 Å), 0.98 eV (1.90 Å) and 0.76 eV (2.46 Å) for bridge, step-edge, kink-O and kink-Mg, respectively. The resulting energy level-alignment is presented in Fig. 8.

The HOMO and LUMO states of the bridge and low-coordinated adsorbed systems and the multilayer phase are macrocycle in character. This allows us to make a direct comparison based on their energy differences. In the case of the kink-Mg, the macrocycle-state, which takes on the charge, is presented separately, lying completely at mid-gap and separated from the rest of the valence band (see Fig. 8). We observe a more pronounced closing of the HOMO–LUMO gap for the step-edge, 2.8 eV, and kink-O, 2.6 eV, adsorption as compared with the bridge site, 3.7 eV, and the crystalline film, 3.4 eV. In addition, a careful selection of a charge donor with a Fermi level lying between the bridge and step-edge/kink-O LUMOs could be used to selectively charge only adsorbates at the low-coordinated sites. However, we do not consider this in more detail here as this is not a relevant scenario for our experiments.

**Fig. 9:**
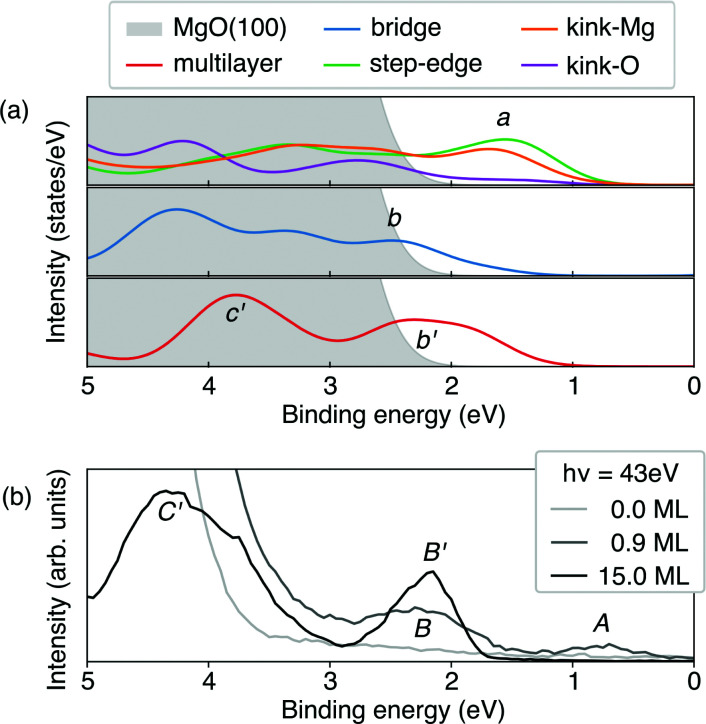
(a) Simulated photoemission spectra of the CoTPP multilayer and CoTPP adsorbed at the MgO surface at different sites. All electronic levels are broadened by a Gaussian function with sigma of 0.3 eV and are merged without considering the spin-polarization. The energy range is adjusted, so as to match the experimental one, by aligning the density of states to the experimental peak B′; (b) experimental UPS spectra for CoTPP as adsorbed on MgO at different coverages.

Based on the level-alignment scheme, we can compare our results with UPS experiments. To this end, we simulated the UPS spectra using the projected density of states (PDOS), where the contribution of each element state was weighed by its photoionization cross-section^32^ for a photon of 45 eV,^97–99^ close to the one used in the experiment.^40^ It is important to note that at such a photon energy, the Co-3d states would be much more pronounced than the corresponding carbon ones, which govern the CoTPP HOMOs. To obtain the overall photoelectron spectrum we assume that the monolayer can be decomposed to individual CoTPP molecules on the investigated adsorption sites, where the surface contribution has been excluded for clarity. The overall photo-emission spectrum for these configurations is shown in Fig. 9a.

Adsorption at the step-edge and kink-Mg leads to a pronounced peak, labelled a, already seen for one adsorbed molecule and higher than its corresponding equivalent in the bridge-CoTPP (see Fig. 9a). The bridge-CoTPP, together with other adsorption sites, contributes mainly to a second peak, b. If we assume that 80% of the molecules occupy a terrace site, similarly to our discussion regarding the XPS spectra, the bridge contribution would appear four times more intense, enhancing thus peak b. This indeed agrees well with the experimental spectrum of the monolayer, where two peaks are observed – one with lower intensity at lower binding energy (peak A) and one with higher intensity at higher binding energy (peak B). Other more intense contributions are expect to remain hidden under the MgO valence band.

In addition, we show the simulated photoemission spectrum of the CoTPP crystal, which represents the contribution from CoTPP multilayer adsorbed on MgO. We observe again two peaks, b′ and c′. The lowest in binding energy (b′) aligns with the second peak in the monolayer (b), whereas the c′ appears in the region with strong MgO contribution. These two peaks can be clearly related to the experimental ones (see Fig. 9b).^40^ Our analysis of the simulated UPS spectra shows that the peaks a, b, and b′ comprise contributions from the macrocycle and c′ contributions from the phenyl rings in agreement with an earlier assignment.^19^ In addition, there are dominant contributions from the Co-3d states. For the CoTPP multilayer, the decomposition is demonstrated in Fig. S6b in the ESI.†

The theoretical spectra reproduce the features observed in the experiment, noteworthy for the multilayer (see Fig. S6a in the ESI†). Still there are short-comings regarding the quantitative separation of peaks (see Fig. 9). Indeed, in the UPS spectrum peak A has a lower binding energy of 0.7 eV compared to B, 2.2 eV, whereas our simulations predicts them to be much closer in energy, 1.6 and 2.5 eV, respectively. Similarly for the multilayer, peaks B′ and C′ are separated by 2.0 eV according to the experimental values, whereas the theory predicts them to stand at 1.5 eV from each other. This could be attributed to the fact that theoretically, the valence states appear more contracted in energy without though compromising the overall description of the system as is the case for the multilayer (see Fig. S6a in the ESI†). Furthermore, the approximations regarding image-potential effects may lead to an underestimation of the peak separation between a and b.

Overall, the theoretical UPS confirms our XPS findings. The new features observed experimentally in the valence band region are to be ascribed to low-coordinated sites, in particular kink-Mg related sites. Also in a very recent work^19^ on the CoTPP adsorption at the oxygen-covered Fe(100) surface pronounced CoTPP-related features in the valence region of UPS spectra were observed for the monolayer. After the adsorption of 4 monolayers the spectra closely resemble ours of the multilayer CoTPP films. There the evolution of the spectra manifests the interaction of CoTPP with the Fe(100)-*p*(1 × 1)O substrate. However, in contrast to our findings, CoTPP adsorption only takes place at the well-ordered surface, as scanning tunneling microscopy experiments and complementary modeling confirm.

### Metal exchange at the surface

In this last section we look into the probability of metal exchange happening with the surface and its effect on the electronic structure. Such a transmetalation process has already been observed for porphyrin-derivatives with surface metals^27,28,100,101^ and by adding metal atoms in gas phase^102^ or at the solid–liquid interfaces.^103^ Such a metal substitution is, however, more demanding than the simple metalation of free-base tetraphenylporphyrins, which is an energetically favourable process.^26,104,105^ A driving factor in the transmetalation is namely the metal-ion radius – the smaller the divalent ions are, the most stable the metalloporphyrin is.^106^ In terms of a trend, it is expected that a smaller ion would substitute a bigger one.

Cobalt and magnesium have radii of similar size, with Mg being 0.07 Å bigger than Co (see Table S9 in the ESI†). Additionally, MgTPP and CoTPP calculations in the gas phase indicate a stronger binding energy by 2.35 eV for the latter.^107^ While these aforementioned factors speak against a transmetalation for CoTPP on MgO, small amounts of MgTPP have been observed during the experimental adsorption.^40^ These were attributed to the metalation of the 10% H_2_TPP impurities present in the CoTPP, without commenting on the possibility of metal exchange. Interestingly though, no characteristic features of H_2_TPP, such as two distinct peaks for the N 1s core-level shifts, were observed, which could be ascribed to the rather small amount of free-base tetraphenylporphyrine. It has been reported that some surfaces may stabilize a metallic centre over another one,^103^ even though no surface atoms were involved in the reaction.

With this in view, we investigate several scenarios in a theoretical experiment. We consider first the possibility for transmetalation *via* a substitution with a surface atom.^101^ This situation is possible, considering that both CoO and MgO crystallize into the rocksalt structure with similar lattice constants (see Table S9 in the ESI†). We constructed thus two models with exchanged Co/Mg ions, where the surface Co is in direct vicinity to the porphyrin metal centre. We establish that the surface transmetalation requires 1.2 eV on the flat MgO(100) and 1.1 eV on the edge of the stepped surface. Such high values are not surprising, considering the high cohesive energy of MgO, 10.3 eV calculated with PBE, and we would thus expect a high activation energy for the process to proceed.

In addition, we look into the transmetalation *via* an adatom^101,105^ where three different scenarios are considered. The reaction is assumed to start with a Mg-adatom and CoTPP adsorbed together on the flat Mg(100) (model A, see Fig. S8a in the ESI†). After the metal substitution we could have either a Co-adatom and MgTPP (model B, see Fig. S8b in the ESI†) or the Co-atom pushed above the macrocycle in the MgTPP^38,104^ (model C, see Fig. S8c in the ESI†).

The energy difference between models A and B amounts to 0.77 eV in favour of the latter (HSE06). This comprises, however, not only the stabilization of the one tetraphenylporphyrin over the other, but also the adatom energy. We ascribe the stabilization of MgTPP to the Co-adatom on the surface, which governs the valence region. The Mg-adatom, conversely, provokes a charge transfer of its two additional electrons to the low-lying empty porphyrin-like states of the molecule (see Fig. S8d in the ESI†).

The peculiar structure of model C also entails filling of the MgTPP empty states, whereas the valence region is governed by hybridized states of the Co and macrocycle. The cobalt maintains a magnetic moment of 1.8 μ, which is lower than the one on the Co-adatom, 2.5 μ, and higher than the one in the CoTPP with 1.0 μ (HSE06). The structure is more favourable than model A and it is 0.32 eV higher in energy than model B. However, this reaction path is largely suppressed due to the expected low abundance of Mg-adatoms at the surface.

## Conclusion

In this work, we investigate the interaction between a Co-substituted tetraphenylporphyrin and an ionic oxide substrate MgO(100). We combine hybrid density-functional theory with a correction scheme based on higher-level methods for the identification of adsorption sites *via* their photoemission fingerprints as observed in our experiments. We established with the help of simulated photoemission spectra that the adsorbate's interaction with surface step-edge and kink-Mg sites explains the distinct features in the valence region of the CoTPP monolayer. We find that transmetalation could be enabled by adatoms, however, at a very low production rate limited by their abundance.

## Conflicts of interest

There are no conflicts to declare.

## Supplementary Material

CP-023-D0CP04859C-s001

## References

[cit1] Borgström M., Blart E., Boschloo G., Mukhtar E., Hagfeldt A., Hammarström L., Odobel F. (2005). J. Phys. Chem. B.

[cit2] Auwärter W., Seufert K., Bischoff F., Ecija D., Vijayaraghavan S., Joshi S., Klappenberger F., Samudrala N., Barth J. V. (2012). Nat. Nanotechnol..

[cit3] Sivalingam Y., Martinelli E., Catini A., Magna G., Pomarico G., Basoli F., Paolesse R., Di Natale C. (2012). J. Phys. Chem. C.

[cit4] Hasobe T. (2013). J. Phys. Chem. Lett..

[cit5] Urbani M., Grätzel M., Nazeeruddin M. K., Torres T. (2014). Chem. Rev..

[cit6] Gottfried J. M. (2015). Surf. Sci. Rep..

[cit7] Maufroy A., Favereau L., Anne F. B., Pellegrin Y., Blart E., Hissler M., Jacquemin D., Odobel F. (2015). J. Mater. Chem. A.

[cit8] Paolesse R., Nardis S., Monti D., Stefanelli M., Di Natale C. (2017). Chem. Rev..

[cit9] Song H., Liu Q., Xie Y. (2018). Chem. Commun..

[cit10] Lukasczyk T., Flechtner K., Merte L. R., Jux N., Maier F., Gottfried J. M., Steinrück H.-P. (2007). J. Phys. Chem. C.

[cit11] Schneider J., Kollhoff F., Schindler T., Bichlmaier S., Bernardi J., Unruh T., Libuda J., Berger T., Diwald O. (2016). J. Phys. Chem. C.

[cit12] Fernández C. C., Wechsler D., Rocha T. C., Steinrück H.-P., Lytken O., Williams F. J. (2019). Surf. Sci..

[cit13] Zamborlini G., Lüftner D., Feng Z., Kollmann B., Puschnig P., Dri C., Panighel M., Di Santo G., Goldoni A., Comelli G. (2017). et al.. Nat. Commun..

[cit14] Buchner F., Warnick K.-G., Wölfle T., Görling A., Steinrück H.-P., Hieringer W., Marbach H. (2009). J. Phys. Chem. C.

[cit15] Hieringer W., Flechtner K., Kretschmann A., Seufert K., Auwärter W., Barth J. V., Görling A., Steinrück H.-P., Gottfried J. M. (2011). J. Am. Chem. Soc..

[cit16] Malcoğlu O. B. and Bockstedte M., in preparation

[cit17] Zoldan V. C., Faccio R., Gao C., Pasa A. A. (2013). J. Phys. Chem. C.

[cit18] Fratesi G., Achilli S., Ugolotti A., Lodesani A., Picone A., Brambilla A., Floreano L., Calloni A., Bussetti G. (2020). Appl. Surf. Sci..

[cit19] Calloni A., Jagadeesh M., Bussetti G., Fratesi G., Achilli S., Picone A., Lodesani A., Brambilla A., Goletti C., Ciccacci F., Duò L., Finazzi M., Goldoni A., Verdini A., Floreano L. (2020). Appl. Surf. Sci..

[cit20] Auwärter W., Seufert K., Klappenberger F., Reichert J., Weber-Bargioni A., Verdini A., Cvetko D., Dell'Angela M., Floreano L., Cossaro A., Bavdek G., Morgante A., Seitsonen A. P., Barth J. V. (2010). Phys. Rev. B: Condens. Matter Mater. Phys..

[cit21] Rangan S., Ruggieri C., Bartynski R., Martínez J. I., Flores F., Ortega J. (2016). J. Phys. Chem. C.

[cit22] Bai Y., Sekita M., Schmid M., Bischof T., Steinrück H.-P., Gottfried J. M. (2010). Phys. Chem. Chem. Phys..

[cit23] Donovan P., Robin A., Dyer M. S., Persson M., Raval R. (2010). Chem. – Eur. J..

[cit24] Schmitt T., Ferstl P., Hammer L., Schneider M. A., Redinger J. (2017). J. Phys. Chem. C.

[cit25] Rojas G., Chen X., Bravo C., Kim J.-H., Kim J.-S., Xiao J., Dowben P. A., Gao Y., Zeng X. C., Choe W., Enders A. (2010). J. Phys. Chem. C.

[cit26] Schneider J., Franke M., Gurrath M., Röckert M., Berger T., Bernardi J., Meyer B., Steinrück H.-P., Lytken O., Diwald O. (2016). Chem. – Eur. J..

[cit27] Marbach H. (2015). Acc. Chem. Res..

[cit28] Diller K., Papageorgiou A. C., Klappenberger F., Allegretti F., Barth J. V., Auwärter W. (2016). Chem. Soc. Rev..

[cit29] Di Filippo G., Classen A., Pöschel R., Fauster T. (2017). J. Chem. Phys..

[cit30] Ishida N., Fujita D. (2012). J. Phys. Chem. C.

[cit31] Martínez J. I., Flores F., Ortega J., Rangan S., Ruggieri C. M., Bartynski R. A. (2017). Phys. Chem. Chem. Phys..

[cit32] Zhang T., Brumboiu I. E., Lanzilotto V., Lüder J., Grazioli C., Giangrisostomi E., Ovsyannikov R., Sassa Y., Bidermane I., Stupar M., de Simone M., Coreno M., Ressel B., Pedio M., Rudolf P., Brena B., Puglia C. (2017). J. Phys. Chem. C.

[cit33] Comanici K., Buchner F., Flechtner K., Lukasczyk T., Gottfried J. M., Steinrück H.-P., Marbach H. (2008). Langmuir.

[cit34] Scudiero L., Barlow D. E., Mazur U., Hipps K. W. (2001). J. Am. Chem. Soc..

[cit35] Flechtner K., Kretschmann A., Steinrück H.-P., Gottfried J. M. (2007). J. Am. Chem. Soc..

[cit36] Gottfried M., Marbach H. (2009). Z. Phys. Chem..

[cit37] Wäckerlin C., Tarafder K., Siewert D., Girovsky J., Hählen T., Iacovita C., Kleibert A., Nolting F., Jung T. A., Oppeneer P. M., Ballav N. (2012). Chem. Sci..

[cit38] Vijayaraghavan S., Auwärter W., Ecija D., Seufert K., Rusponi S., Houwaart T., Sautet P., Bocquet M.-L., Thakur P., Stepanow S., Schlickum U., Etzkorn M., Brune H., Barth J. V. (2015). ACS Nano.

[cit39] Ballav N., Wäckerlin C., Siewert D., Oppeneer P. M., Jung T. A. (2013). J. Phys. Chem. Lett..

[cit40] Franke M., Wechsler D., Tariq Q., Röckert M., Zhang L., Kumar Thakur P., Tsud N., Bercha S., Prince K., Lee T.-L., Steinrück H.-P., Lytken O. (2017). Phys. Chem. Chem. Phys..

[cit41] Zhao H., Yang Y., Shu X., Wang Y., Ran Q. (2018). Adv. Colloid Interface Sci..

[cit42] Hedin L. (1965). Phys. Rev..

[cit43] van Setten M. J., Caruso F., Sharifzadeh S., Ren X., Scheffler M., Liu F., Lischner J., Lin L., Deslippe J. R., Louie S. G., Yang C., Weigend F., Neaton J. B., Evers F., Rinke P. (2015). J. Chem. Theory Comput..

[cit44] Neaton J. B., Hybertsen M. S., Louie S. G. (2006). Phys. Rev. Lett..

[cit45] Thygesen K. S., Rubio A. (2009). Phys. Rev. Lett..

[cit46] Chen Y., Tamblyn I., Quek S. Y. (2017). J. Phys. Chem. C.

[cit47] Kronik L., Neaton J. B. (2016). Annu. Rev. Phys. Chem..

[cit48] Migani A., Mowbray D. J., Zhao J., Petek H., Rubio A. (2014). J. Chem. Theory Comput..

[cit49] Quek S. Y., Venkataraman L., Choi H. J., Louie S. G., Hybertsen M. S., Neaton J. B. (2007). Nano Lett..

[cit50] Quek S. Y., Choi H. J., Louie S. G., Neaton J. B. (2009). Nano Lett..

[cit51] Souza A. M., Rungger I., Pemmaraju C. D., Schwingenschloegl U., Sanvito S. (2013). Phys. Rev. B: Condens. Matter Mater. Phys..

[cit52] Egger D. A., Liu Z.-F., Neaton J. B., Kronik L. (2015). Nano Lett..

[cit53] Ma J., Liu Z.-F., Neaton J. B., Wang L.-W. (2016). Appl. Phys. Lett..

[cit54] Hollerer M., Lüftner D., Hurdax P., Ules T., Soubatch S., Tautz F. S., Koller G., Puschnig P., Sterrer M., Ramsey M. G. (2017). ACS Nano.

[cit55] Perdew J. P., Burke K., Ernzerhof M. (1996). Phys. Rev. Lett..

[cit56] Grimme S., Antony J., Ehrlich S., Krieg H. (2010). J. Chem. Phys..

[cit57] Marom N., Hod O., Scuseria G. E., Kronik L. (2008). J. Chem. Phys..

[cit58] Krukau A. V., Vydrov O. A., Izmaylov A. F., Scuseria G. E. (2006). J. Chem. Phys..

[cit59] Dudarev S. L., Botton G. A., Savrasov S. Y., Humphreys C. J., Sutton A. P. (1998). Phys. Rev. B: Condens. Matter Mater. Phys..

[cit60] Madura P., Scheidt R. (1976). Inorg. Chem..

[cit61] MgO occurs naturally as the mineral periclase. It has a cubic unit cell in *Fm*3̄*m* space group, where the lattice constant is 4.216 (Å)^108^ or 4.212 (Å).^109^ The ionic arrangement is rock-salt, where the non-polar surface plane is along (100). Our fit of bulk unit cells to the Birch–Murnaghan equation of state determined a lattice constant of 4.239 Å in close agreement with the experiment.

[cit62] Kresse G., Furthmüller J. (1996). Comput. Mater. Sci..

[cit63] Kresse G., Furthmüller J. (1996). Phys. Rev. B: Condens. Matter Mater. Phys..

[cit64] Blöchl P. E. (1994). Phys. Rev. B: Condens. Matter Mater. Phys..

[cit65] Kresse G., Joubert D. (1999). Phys. Rev. B: Condens. Matter Mater. Phys..

[cit66] Malcoğlu O. B., Bechis I., Bockstedte M. (2020). Phys. Chem. Chem. Phys..

[cit67] Schintke S., Messerli S., Pivetta M., Patthey F., Libioulle L., Stengel M., De Vita A., Schneider W.-D. (2001). Phys. Rev. Lett..

[cit68] Momma K., Izumi F. (2011). J. Appl. Crystallogr..

[cit69] Yokoyama T., Yokoyama S., Kamikado T., Okuno Y., Mashiko S. (2001). Nature.

[cit70] Valeri S., Altieri S., del Pennino U., di Bona A., Luches P., Rota A. (2002). Phys. Rev. B: Condens. Matter Mater. Phys..

[cit71] Sterrer M., Fischbach E., Risse T., Freund H.-J. (2005). Phys. Rev. Lett..

[cit72] Savio L., Smerieri M., Orzelli A., Vattuone L., Rocca M., Finocchi F., Jupille J. (2010). Surf. Sci..

[cit73] Wollschläger J., Viernow J., Tegenkamp C., Erdös D., Schröder K., Pfnür H. (1999). Appl. Surf. Sci..

[cit74] Ouvrard A., Niebauer J., Ghalgaoui A., Barth C., Henry C. R., Bourguignon B. (2011). J. Phys. Chem. C.

[cit75] Pal J., Smerieri M., Celasco E., Savio L., Vattuone L., Rocca M. (2014). Phys. Rev. Lett..

[cit76] Heiz U., Schneider W.-D. (2000). J. Phys. D: Appl. Phys..

[cit77] Pacchioni G., Pescarmona P. (1998). Surf. Sci..

[cit78] van Gog H., van Huis M. A. (2019). J. Phys. Chem. C.

[cit79] Finocchi F., Geysermans P., Bourgeois A. (2012). Phys. Chem. Chem. Phys..

[cit80] Henkelman G., Arnaldsson A., Jónsson H. (2006). Comput. Mater. Sci..

[cit81] Schmid M., Zirzlmeier J., Steinrück H.-P., Gottfried J. M. (2011). J. Phys. Chem. C.

[cit82] Sterrer M., Heyde M., Novicki M., Nilius N., Risse T., Rust H.-P., Pacchioni G., Freund H.-J. (2006). J. Phys. Chem. B.

[cit83] Köhler L., Kresse G. (2004). Phys. Rev. B: Condens. Matter Mater. Phys..

[cit84] Janak J. F. (1978). Phys. Rev. B: Solid State.

[cit85] Slater J. C., in Advances in Quantum Chemistry, ed. P.-O. Löwdin, Academic Press, 1972, vol. 6, pp. 1–92

[cit86] Pueyo Bellafont N., Viñes F., Hieringer W., Illas F. (2017). J. Comput. Chem..

[cit87] Torelli P., Giordano L., Benedetti S., Luches P., Annese E., Valeri S., Pacchioni G. (2009). J. Phys. Chem. C.

[cit88] Thomele D., Bourret G. R., Bernardi J., Bockstedte M., Diwald O. (2017). Angew. Chem., Int. Ed..

[cit89] Biller A., Tamblyn I., Neaton J. B., Kronik L. (2011). J. Chem. Phys..

[cit90] Liu Z.-F., Egger D. A., Refaely-Abramson S., Kronik L., Neaton J. B. (2017). J. Chem. Phys..

[cit91] Kronik L., Kümmel S. (2018). Adv. Mater..

[cit92] Gunnarsson O., Lundqvist B. I. (1976). Phys. Rev. B: Solid State.

[cit93] Garcia-Lastra J. M., Rostgaard C., Rubio A., Thygesen K. S. (2009). Phys. Rev. B: Condens. Matter Mater. Phys..

[cit94] Li Y., Lu D., Galli G. (2009). J. Chem. Theory Comput..

[cit95] Yu M., Doak P., Tamblyn I., Neaton J. B. (2013). J. Phys. Chem. Lett..

[cit96] Roychoudhury S., Motta C., Sanvito S. (2016). Phys. Rev. B.

[cit97] Yeh J., Lindau I. (1985). At. Data Nucl. Data Tables.

[cit98] Yeh J., Atomic Calculation of Photoionization Cross-sections and Asymmetry Parameters, Gordon & Breach Science, Publishers, 1993

[cit99] Atomic Calculation of Photoionization Cross-Sections and Asymmetry Parameters, https://vuo.elettra.eu/services/elements/WebElements.html, accessed: July 2019

[cit100] Doyle C. M., Cunniffe J. P., Krasnikov S. A., Preobrajenski A. B., Li Z., Sergeeva N. N., Senge M. O., Cafolla A. A. (2014). Chem. Commun..

[cit101] Shen K., Narsu B., Ji G., Sun H., Hu J., Liang Z., Gao X., Li H., Li Z., Song B., Jiang Z., Huang H., Wells J. W., Song F. (2017). RSC Adv..

[cit102] Wang C., Fan Q., Han Y., Martínez J. I., Martín-Gago J. A., Wang W., Ju H., Gottfried J. M., Zhu J. (2016). Nanoscale.

[cit103] Franke M., Marchini F., Jux N., Steinrück H.-P., Lytken O., Williams F. J. (2016). Chem. – Eur. J..

[cit104] Shubina T. E., Marbach H., Flechtner K., Kretschmann A., Jux N., Buchner F., Steinrück H.-P., Clark T., Gottfried J. M. (2007). J. Am. Chem. Soc..

[cit105] Goldoni A., Pignedoli C. A., Di Santo G., Castellarin-Cudia C., Magnano E., Bondino F., Verdini A., Passerone D. (2012). ACS Nano.

[cit106] Barnes J. W., Dorough G. D. (1950). J. Am. Chem. Soc..

[cit107] Liao M.-S., Scheiner S. (2001). J. Chem. Phys..

[cit108] Broser I., Broser R., Finkenrath H., Galazka R., Gumlich H., Hoffmann A., Kossut J., Mollwo E., Nelkowski H., Nimtz G., *et al.*, Physics of II-VI and I-VII Compounds, Semimagnetic Semiconductors/Physik Der II-VI und I-VII-Verbindungen, Semimagnetische Halbleiter Elemente, Springer, 1982

[cit109] Hazen R. M. (1976). Am. Mineral..

