# Supplementary material for: Morphology dependent interaction between Co(ii)-tetraphenylporphyrin and the MgO(100) surface
Source: Phys Chem Chem Phys. 2021 Jan 4;23(3):2105–16. doi: 10.1039/d0cp04859c (PMC8431532; doi:10.1039/d0cp04859c)
Supplement: CP-023-D0CP04859C-s001 [file CP-023-D0CP04859C-s001.pdf]

This journal is © the Owner Societies 2021

Electronic Supplementary Information for

Morphology Dependent Interaction between Co(II)-tetraphenylporphyrin and the MgO(100) surface

by

Silviya Ninova, Osman Barış Malcıoğlu, Philipp Auburger, Matthias Franke, Ole Lytken, Hans-Peter Steinrück, Michel Bockstedte

S1 CoTPP in gas phase

The CoTPP optimized geometry remains close to the experimental one as extracted from the crystal with minimal differences between the different functionals (see Table S1). The strongest deviations are observed for the angles, in particular the out-of-plane one.

Table S1 Geometry parameters for the CoTPP optimized in gas phase with different functionals. The average Co-N bond length (in Å) is reported. The negative sign of the ϕ angles corresponds to the phenyl rings tilted downwards with respect to the macrocycle plane. The experimental values are as extracted from the crystal structure. [1]

Functional	d_{Co-N} (Å)	θ_1 (°)	θ_2 (°)	θ_3 (°)	θ_4 (°)	ϕ_1 (°)	ϕ_2 (°)	ϕ_3 (°)	ϕ_4 (°)
PBE+D3	1.958	63.7	63.6	63.6	63.6	-9.2	9.1	-9.1	9.1
PBE0	1.957	63.7	63.5	63.6	63.6	-9.3	9.3	-9.3	9.3
HSE06	1.958	63.4	63.3	63.4	63.4	-9.4	9.4	-9.3	9.3
B3LYP	1.971	63.9	63.8	63.9	63.9	-9.3	9.3	-9.3	9.3
Experiment [1]	1.949	80.0	80.0	80.0	80.0	-14.1	14.1	-14.1	14.1

As opposed to the geometry, the electronic structure undergoes significant changes for the different functionals, with PBE being wrong in the valence region (see Figure S1). We thus adopt a computational protocol, where the geometry is relaxed within the more cost-efficient PBE+D3, and the electronic structure is determined in a subsequent calculation with the HSE06 hybrid functional or GW0 for the level alignment.

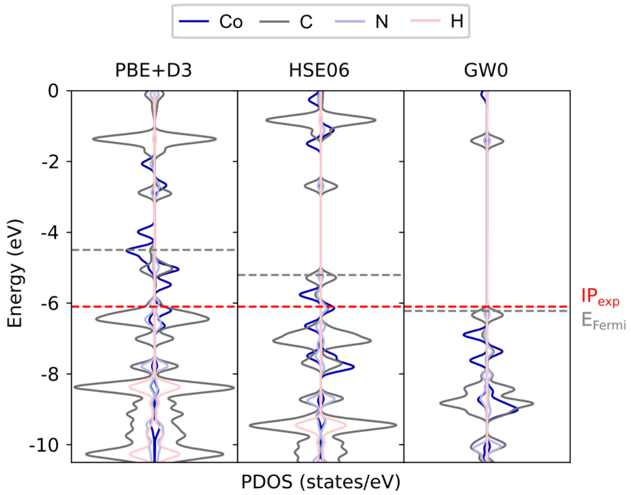

Figure S1 PDOS of the CoTPP in gas phase calculated with PBE+D3 (left), HSE06 (middle) and GW0 (right). Alignment with respect to the vacuum is applied, so as to compare with the experimental ionization potential (IP_{exp} [2]). A convolution with Gaussian functions was applied on all PDOS with a width of 0.1 eV.

S2 CoTPP@MgO(100)

S2.1 Single CoTPP

We observe some geometry changes to the macrocycle or phenyl rings compared to the isolated structure. Indeed, it has been proved both experimentally and theoretically that the phenyl rings can bend so as to maximize the contact of the macrocycle with the surface.[3–5]

Table S2 Geometry parameters for the CoTPP optimized on the MgO(100) in comparison with the molecule in the gas phase. The average Co-N bond length (in Å) is reported with the standard deviation in parentheses. All angles are in °. The negative sign of the ϕ angles corresponds to the phenyl rings tilted downwards with respect to the macrocycle plane.

Site	d_{Co-N}	θ_1	θ_2	θ_3	θ_4	ϕ_1	ϕ_2	ϕ_3	ϕ_4
gas phase	1.958 (0.000)	63.7	63.6	63.6	63.6	-9.2	9.1	-9.1	9.1
bridge	1.957 (0.007)	43.3	49.2	48.4	43.2	6.7	6.6	6.3	6.9
bridge-r45	1.959 (0.004)	41.8	36.0	37.0	41.5	8.7	2.8	1.1	7.5
ontop	1.974 (0.008)	46.1	49.6	47.1	47.8	7.2	11.0	8.8	10.0
ontop-r45	1.971 (0.008)	45.2	51.3	47.1	49.6	9.0	13.6	11.2	11.8
step-edge	1.976 (0.015)	41.8	38.6	31.4	85.3	-9.2	-5.6	1.8	-9.5
kink-Mg	1.941 (0.006)	45.0	50.6	36.8	40.7	14.7	1.5	-10.9	2.5
kink-O	1.993 (0.024)	43.7	55.2	44.1	42.3	9.0	-3.1	-16.2	-2.4

Table S3 Literature overview on the simulated CoTPP adsorption on different substrates.

Surface	Functional	$d_{Co-surf}$ (Å)	Adsorption site	Distortion	Ref.
Cu(110)	PW91-vdW-DF	2.2	short-bridge for domains	flat with tilting distortions	4
Cu(110)	PW91-vdW-DF	2.4	long-bridge for individual	non-planar, saddle-shape conformation	4
Cu(110)	PBE	2.87	short-bridge	saddle-shape conformation	6
Cu_3N-Cu(110)	PBE	2.72	on-top N	saddle-shape conformation	6
Ag(111)	PBE-vdW	3.13[a]	on-top	flat due to the lacking 4 phenyl rings	7
Ag(111)	LDA		fcc and hcp hollow	saddle-shape conformation	8
Fe(100)-p(1×1)O	vdW-DF-c09	3.28	on-top Fe $(5\times5)R37^\circ$		12
		3.18	on-top O $(5\times5)R37^\circ$		12

[a] The distance is evaluated with respect to the surface. The Co-Ag bond with the underlying silver atom is 2.83 (Å).

S2.2 Monolayer CoTPP

Single-molecule adsorption neglects possible Van der Waals interactions between the neighbouring molecules in a monolayer. This is especially true for the case of tetraphenylporphyrins where a great part of the interaction is between the phenyl rings. We, therefore, investigated the geometry and electronic structure of a CoTPP monolayer on the flat MgO(100) surface.

The model consists of a reduced slab width, $(21.195\times21.195\times27.000)$ Å^3, so as to take into advantage the symmetry of the adsorbed CoTPPs (see Figure S2a). The CoTPPs were positioned on the surface, maintaining the structure of the crystal, where each phenyl ring faces another phenyl ring from another CoTPP. In a first approximation, we investigated only the ontop adsorption site.

The monolayer surface positioning resembles that of the isolated CoTPP, which enables us to make a direct comparison (see Table S4). The monolayer Co-ion lies slightly further away from the MgO surface, forming shorter bonds with the porphyrin Nitrogens. The phenyl rings are more flattened with respect to the macrocycle and the surface, so as to enhance the interactions with the neighbouring molecules and the surface at the same time. The angles are indeed more than doubled with respect to the crystal structure. Additionally, if one assumes that the CoTPPs occupy the same adsorption sites on the surface, this would induce a small strain, 0.15%, on the monolayer with respect to the crystal structure.

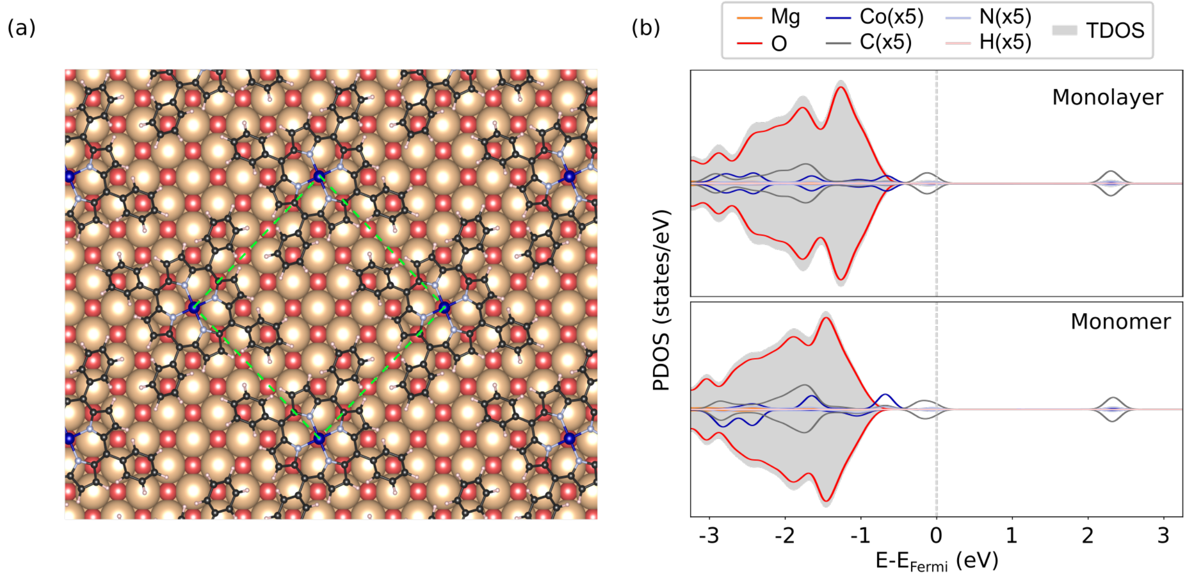

Figure S2 (a) Geometry of the CoTPP monolayer, where the green square indicates the crystal cell; (b) PDOS of the monolayer and a single adsorbed CoTPP, calculated with HSE06 α=0.25.

Table S4 Geometry parameters for the CoTPP at the ontop site on MgO(100) and in the crystal. In the case of the Co-N bond lengths, the average value is reported together with the standard deviation in parentheses. All energies are in eV, distances in Å and angles in °. The negative sign of the ϕ angles corresponds to the phenyl rings tilted downwards with respect to the macrocycle plane. The monolayer contains two inequivalent CoTPP monomers (monolayer_1, monolayer_2), the parameters of each are presented.

	d_{Co-MgO}	d_{Co-O}	d_{Co-N}	θ_1	θ_2	θ_3	θ_4	ϕ_1	ϕ_2	ϕ_3	ϕ_4
single	3.21	3.11	1.974 (0.008)	46.1	49.6	47.1	47.8	7.2	11.0	8.8	10.0
monolayer_1	3.30	3.21	1.962 (0.004)	36.7	33.6	31.6	35.3	3.8	4.4	3.2	4.3
monolayer_2	3.30	3.21	1.962 (0.004)	36.0	34.1	32.3	34.8	4.5	3.6	2.5	5.1
crystal	-	-	1.948 (0.000)	75.4	75.4	75.4	75.4	13.9	-13.9	13.9	-13.9

S3 DFT+U

We avoid the self-interaction error in the electronic structure of CoTPP by working with a hybrid functional (see Section S1). Given the large system we investigate, it would be computationally highly demanding to relax the geometry with HSE06. For this reason, we tested the DFT+U method[9] for the CoTPP adsorbed on the surfaces in terms of geometries and energy differences.

Simulations on Co-porphyrins work with U-J=3 eV[10,11 12,13], whereas the linear-response approach determines a value of 6.9 eV for Co-phthalocyanine (only the metal ion and the pyrrole N atoms were considered)[14]. Other studies on Co-phthalocyanine suggest U(Co)=4 eV and J(Co)=1 eV.[15–18]

In the present study, we look at how a range of U values (0-8 eV) affect the ionization potential, comparing with the experimental value. The HOMO position converges already at U=3 eV, because at this point the frontal occupied orbital is not a transition-metal-only state as with PBE but a macrocycle one. The electronic structure, however, begins to resemble the one of HSE06 only at U=5 eV, which is the value we used for the geometry relaxation.

The electronic structure of the CoTPP on MgO with PBE+U, however, differs from the one with the hybrid functional HSE06 (see Figure S4). In all studied adsorbed cases, the CoTPP valence states with PBE+U lie in same region as the substrate, whereas using the hybrid functional some molecular states appear above the valence band edge. The energy difference between the bridge and ontop-r45 adsorption sites, though, is maintained at 0.1 eV. The geometry changes are also small (see Table S5), similarly to other studies on CoTPP which make use of DFT+U.[12,13]

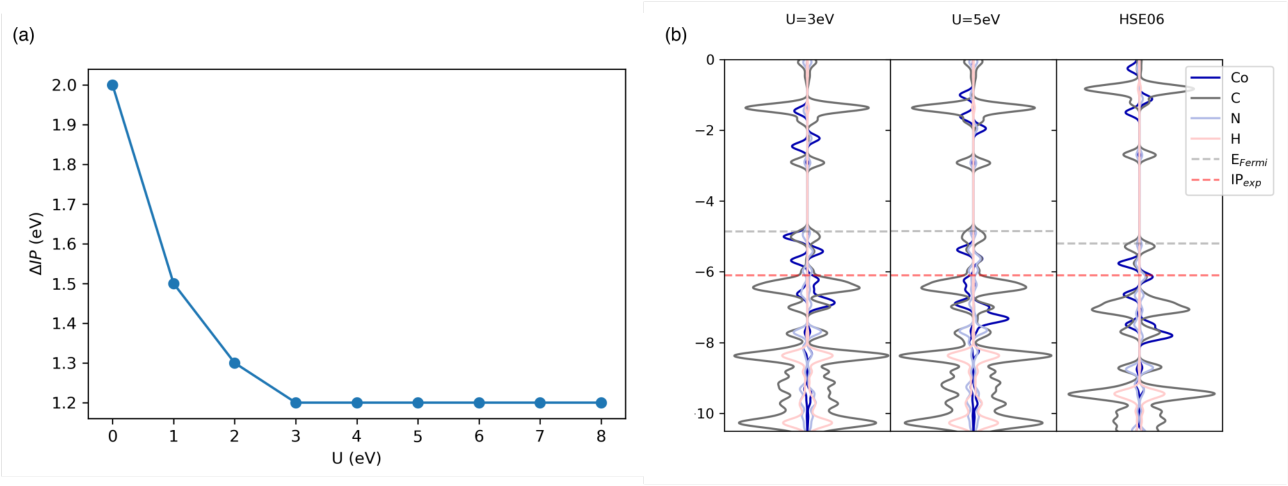

Figure S3 (a) Energy difference (ΔIP) between the calculated HOMO (ε_{HOMO}) and the experimental ionization potential[2]; (b) CoTPP electronic structure at two U values compared with the HSE06 one.

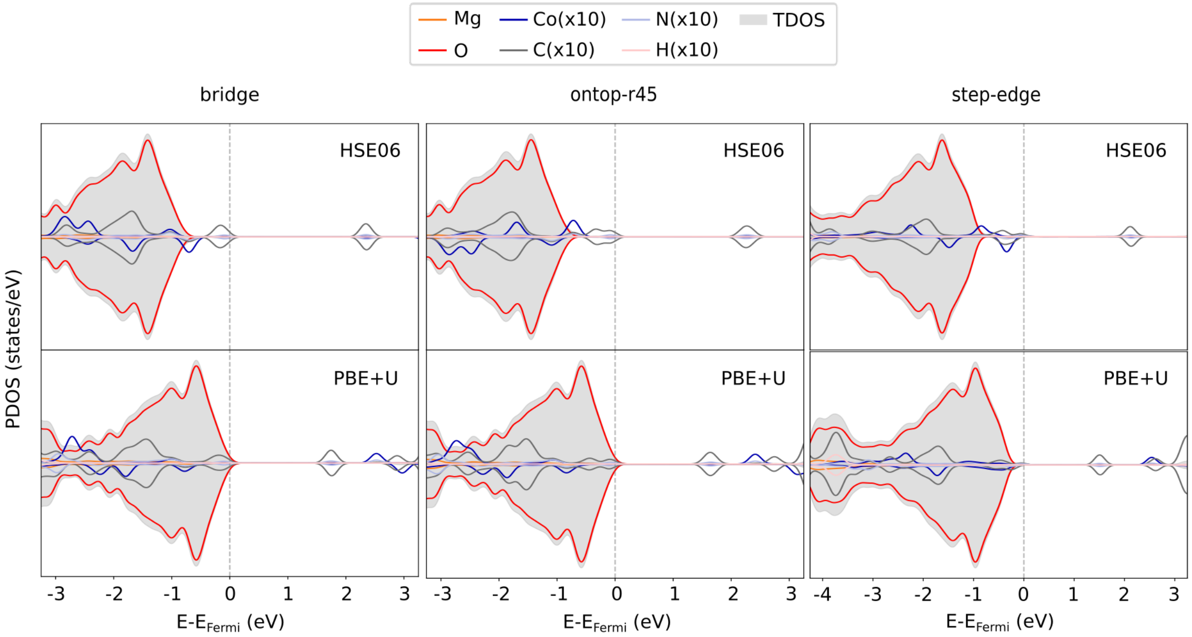

Figure S4 Electronic structure for the CoTPP at bridge, ontop-r45 and step-edge adsorption sites obtained with HSE06 (top) and PBE+U (bottom).

Table S5 Geometry parameters for the CoTPP optimized on the MgO(100) with PBE+U. The average Co-N bond length (in Å) is reported with the standard deviation in parentheses. All angles are in °.

Site	functional	d_{Co-O}	d_{Co-N}	θ_1	θ_2	θ_3	θ_4	ϕ_1	ϕ_2	ϕ_3	ϕ_4
bridge	PBE		1.957 (0.007)	43.3	49.2	48.4	43.2	6.7	6.6	6.3	6.9
	PBE+U		1.985 (0.008)	45.0	47.8	47.5	45.1	6.7	6.4	6.2	6.8
ontop-r45	PBE	3.12	1.971 (0.008)	45.2	51.3	47.1	49.6	9.0	13.6	11.2	11.8
	PBE+U	3.17	1.996 (0.009)	45.8	50.8	47.1	48.8	9.3	13.0	10.9	11.3
step-edge	PBE	2.17	1.976 (0.015)	41.8	38.6	31.4	85.3	-9.2	-5.6	1.8	-9.5
	PBE+U	2.18	2.013 (0.015)	41.4	37.9	31.8	85.6	-8.3	-4.8	1.8	-9.5

S4 Crystal electronic structure and level alignment

CoTPP crystallizes in a tetragonal structure $I\bar{4}2d$[1,19] (see Figure S5a). We optimized the crystal geometry using the PBE+D3 functional. Our optimized geometry is in good agreement with the reported experimental ones (see Table S6).

Table S6 Geometry parameters for the CoTPP molecular crystal as reported in the literature and optimized in this work (PBE+D3).

	a (Å)	b (Å)	c (Å)
exp (100 K)[19]	14.980	14.980	13.704
exp (293 K)[20]	15.062	15.062	13.954
this work	14.964	14.964	13.445

Correct description of the electronic structure of molecular crystals can be achieved within the GW-approximation. Indeed, a gap renormalization is observed in the crystal as compared to the molecule in gas phase (see Figure S5). Small changes in the GW0 density of states are present with respect to those obtained with HSE06. In addition, we find a good correspondence between the multilayer GW0 simulated UPS spectra and the experimental one (see Figure S6).

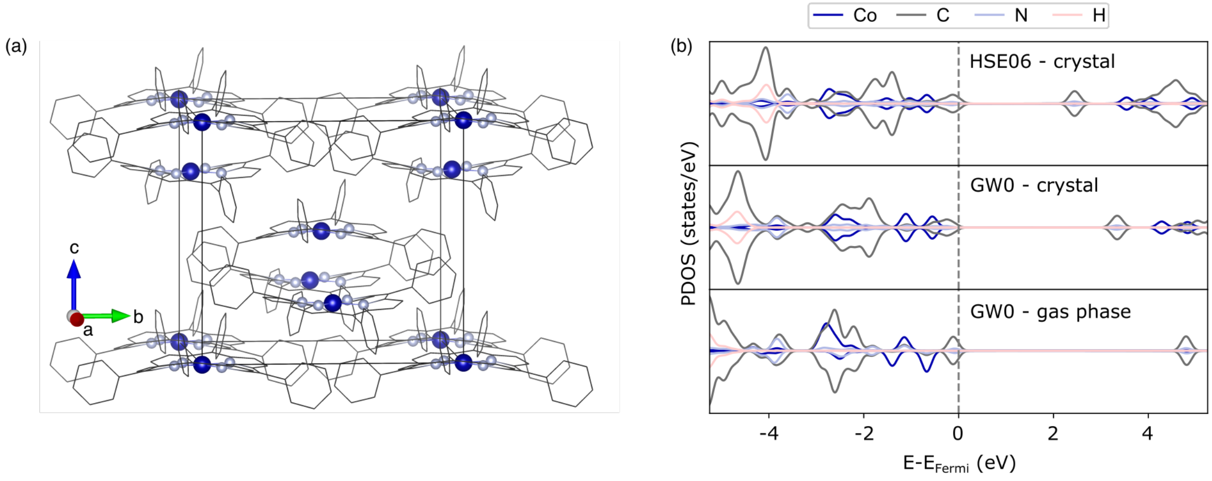

Figure S5 Geometry and PDOS of the CoTPP in the crystal. (a) Geometry as obtained with PBE+D3; (b) PDOS as obtained with with HSE06 (top) and GW0 (middle), compared with the CoTPP in gas phase with GW0 (bottom). A Gaussian σ of 0.1 eV was used for the convolution of the PDOS.

Direct comparison between the energy levels of CoTPP crystal/multilayer and CoTPP@MgO can be achieved when a common energy reference is present, such as the vacuum level for instance. While the vacuum level is determined in a straightforward manner for slab calculations, the procedure for crystals require a few additional steps. To this end, we adopted the approach outlined by Malcıoğlu *et al.*[21], where the crystal electrostatic potential is evaluated in a fully periodic cell and in a slab. The potential energy in the bulk can be related to that in the middle of the slab, which can be later reported with respect to the vacuum level from the slab calculation (see Figure S7). Overall, the required shift (ΔV) corresponds to 5.37 eV.

The crystal geometry was optimized in a separate calculation, starting from the experimental structure.[1] The level alignment was performed on a simulation cell doubled along the z-axis - (14.964×14.964×26.890 Å^3), corresponding thus to 8 CoTPP layers in the direction. In the slab calculation ∼13 Å vacuum was added.

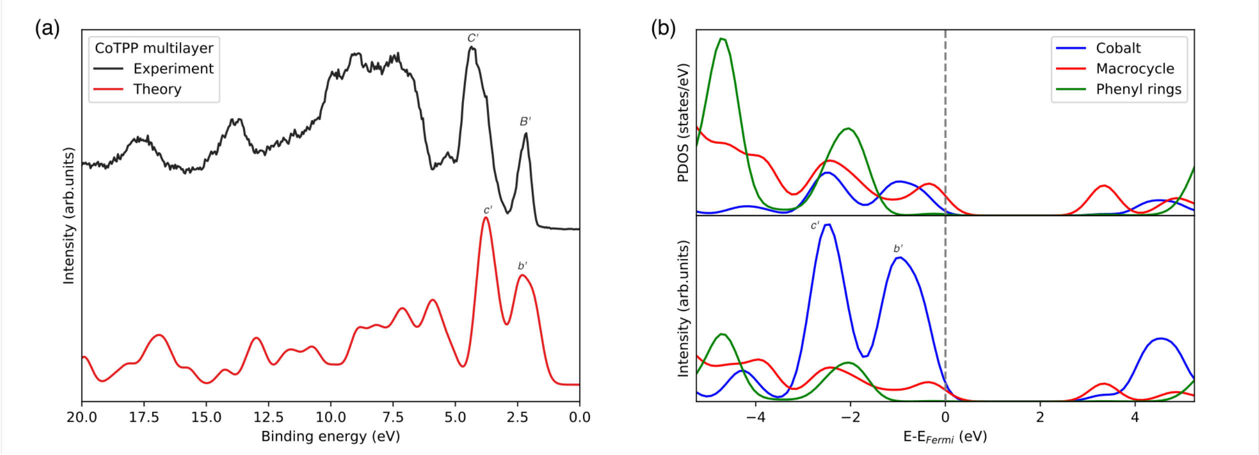

Figure S6 (a) Comparison between the experimental UPS spectrum of the CoTPP multilayer (15 ML) and the simulated one using crystal state calculated with GW0 PDOS. The theoretical spectrum has been shifted so as to match the experimental peak with lowest binding energy. (b) Decomposition of the PDOS (upper panel) and the simulated UPS spectrum into contributions from the cobalt, porphyrin macrocycle and the phenyl rings. The peaks in the upper valence region are labeled according to Figure 9 in the main text. A Gaussian σ of 0.3 eV was used for the convolution of the states.

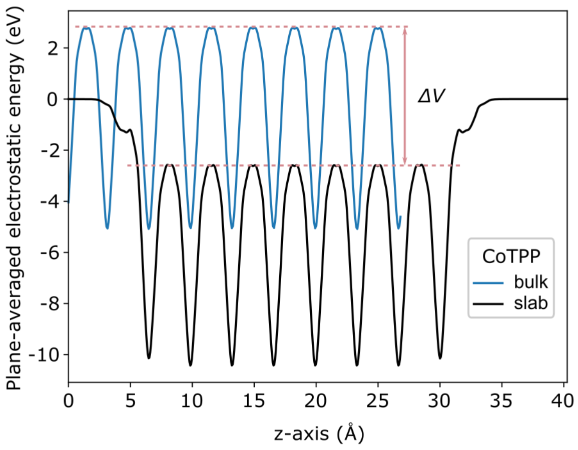

Figure S7 Electrostatic energy for the CoTPP crystal as bulk and slab. For the latter, the values were aligned with respect to the vacuum level. ΔV is the shift used to relate the bulk potential to the vacuum level (5.37 eV).

Table S7 The values used to construct Figure 8 in the main text are summarized in the table below in the format (IP/EA).

System	HSE06	GW0	HSE06+Σ	Exp.
MgO(100) surface	-6.2 / -1.1	-7.1 /-0.4	-	-7.0 / -0.8 [22]
CoTPP gas phase	-5.2 / -2.7	-6.3 / -1.4	-	-6.1 [2] / -
CoTPP@MgO bridge	-5.3 / -2.9	-	-5.9 / -2.2	-
CoTPP@MgO step-edge	-5.1 / -2.9	-	-5.3 / -2.5	-
CoTPP@MgO kink-O	-5.4 / -3.2	-	-5.5 / -2.9	-
CoTPP@MgO kink-Mg	-4.7 / -2.5	-	-5.2 / -1.8	-
CoTPP multilayer	-5.3 / -2.8	-5.6 / -2.2	-	-

S5 Core-level shifts

The Co 2p binding energies and respective shifts, as calculated with the Janak-Slater transition-state (JS) approximation[23,24], are reported in Table S8. The theoretical simulation gives reliable shifts, but may not give exact binding ener-

gies.[25]

Table S8 Absolute values for the Co 2p binding energies and core-level shifts (in eV) as estimated in the Janak-Slater transition-state (JS) approximation.

	bridge	top	step-edge	kink-Mg	kink-O	multilayer
Binding energy	787.1	787.0	786.2	788.3	784.8	786.9
Shift	0.0	-0.1	-0.9	-2.3	1.2	-0.2

S6 Surface Transmetalation

Table S9 Comparison between the crystal, ionic and lattice radii[26] of cobalt and magnesium, so as to evaluate the possibility for a spontaneous transmetalation.

Element	Spin	Crystal radius (Å)	Ionic radius (Å)	Oxide lattice constant (Å)
Co(II)	HS	0.89	0.75	4.259[27]
	LS	0.79	0.65	
Mg(II)	-	0.86	0.72	4.212[28]

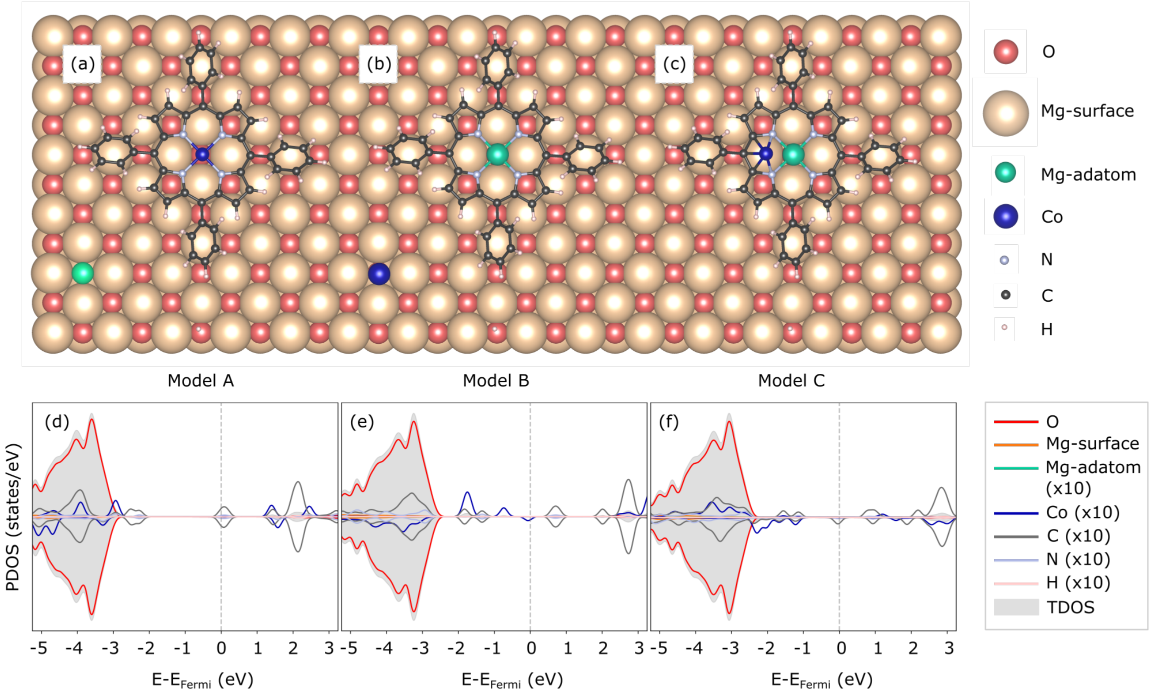

Figure S8 Possible initial (A) or final (B and C) models for the transmetalation process. Their relaxed geometries (a-c) and electronic structure obtained with HSE06 α=0.25 and a Gaussian convolution of 0.1 eV (d-f) are presented.

References

[1] P. Madura and R. Scheidt, *Inorganic Chemistry*, 1976, **15**, 3182–3184.

[2] Y. Nakato, K. Abe and H. Tsubomura, *Chemical Physics Letters*, 1976, **39**, 358 – 360.

[3] A. Weber-Bargioni, W. Auwärter, F. Klappenberger, J. Reichert, S. Lefrançois, T. Strunskus, C. Wöll, A. Schiffrin, Y. Pennec and J. V. Barth, *ChemPhysChem*, 2008, **9**, 89–94.

[4] P. Donovan, A. Robin, M. S. Dyer, M. Persson and R. Raval, *Chemistry – A European Journal*, 2010, **16**, 11641–11652.

[5] G. Zamborlini, D. Lüftner, Z. Feng, B. Kollmann, P. Puschnig, C. Dri, M. Panighel, G. Di Santo, A. Goldoni, G. Comelli *et al.*, *Nature communications*, 2017, **8**, 335.

[6] V. C. Zoldan, R. Faccio, C. Gao and A. A. Pasa, *The Journal of Physical Chemistry C*, 2013, **117**, 15984–15990.

[7] W. Hieringer, K. Flechtner, A. Kretschmann, K. Seufert, W. Auwärter, J. V. Barth, A. Görling, H.-P. Steinrück and J. M. Gottfried, *Journal of the American Chemical Society*, 2011, **133**, 6206–6222.

[8] W. Auwärter, K. Seufert, F. Klappenberger, J. Reichert, A. Weber-Bargioni, A. Verdini, D. Cvetko, M. Dell'Angela, L. Floreano, A. Cossaro, G. Bavdek, A. Morgante, A. P. Seitsonen and J. V. Barth, *Physical Review B*, 2010, **81**, 245403.

[9] S. L. Dudarev, G. A. Botton, S. Y. Savrasov, C. J. Humphreys and A. P. Sutton, *Physical Review B*, 1998, **57**, 1505–1509.

[10] C. Wäckerlin, K. Tarafder, D. Siewert, J. Girovsky, T. Hählen, C. Iacovita, A. Kleibert, F. Nolting, T. A. Jung, P. M. Oppeneer and N. Ballav, *Chemical Science*, 2012, **3**, 3154–3160.

[11] C. Wäckerlin, P. Maldonado, L. Arnold, A. Shchyrba, J. Girovsky, J. Nowakowski, M. E. Ali, T. Hählen, M. Baljozovic, D. Siewert, A. Kleibert, K. Müllen, P. M. Oppeneer, T. A. Jung and N. Ballav, *Chemicam Communications*, 2013, **49**, 10736–10738.

[12] A. Calloni, M. Jagadeesh, G. Bussetti, G. Fratesi, S. Achilli, A. Picone, A. Lodesani, A. Brambilla, C. Goletti, F. Ciccacci, L. Duò, M. Finazzi, A. Goldoni, A. Verdini and L. Floreano, *Applied Surface Science*, 2020, **505**, 144213.

[13] G. Fratesi, S. Achilli, A. Ugolotti, A. Lodesani, A. Picone, A. Brambilla, L. Floreano, A. Calloni and G. Bussetti, *Applied Surface Science*, 2020, **530**, 147085.

[14] I. E. Brumboiu, S. Haldar, J. Lüder, O. Eriksson, H. C. Herper, B. Brena and B. Sanyal, *The Journal of Physical Chemistry A*, 2019, **123**, 3214–3222.

[15] J. Zhou and Q. Sun, *Journal of the American Chemical Society*, 2011, **133**, 15113–15119.

[16] A. Mugarza, R. Robles, C. Krull, R. Korytár, N. Lorente and P. Gambardella, *Physical Review B*, 2012, **85**, 155437.

[17] S. Lach, A. Altenhof, K. Tarafder, F. Schmitt, M. E. Ali, M. Vogel, J. Sauther, P. M. Oppeneer and C. Ziegler, *Advanced Functional Materials*, 2012, **22**, 989–997.

[18] B.-Q. Song, L.-D. Pan, S.-X. Du and H.-J. Gao, *Chinese Physics B*, 2013, **22**, 096801.

[19] E. D. Stevens, *Journal of the American Chemical Society*, 1981, **103**, 5087–5095.

[20] P. Madura and R. Scheidt, *Inorganic Chemistry*, 1976, **15**, 3182–3184.

[21] O. B. Malcıoğlu, I. Bechis and M. Bockstedte, *Physical Chemistry Chemical Physics*, 2020, **22**, 3825–3830.

[22] S. Schintke, S. Messerli, M. Pivetta, F. Patthey, L. Libioulle, M. Stengel, A. De Vita and W.-D. Schneider, *Physical Review Letters*, 2001, **87**, 276801.

[23] J. F. Janak, *Physical Review B*, 1978, **18**, 7165–7168.

[24] J. C. Slater, *Advances in Quantum Chemistry*, Academic Press, 1972, vol. 6, pp. 1 – 92.

[25] L. Köhler and G. Kresse, *Physical Review B*, 2004, **70**, 165405.

[26] R. D. Shannon, *Acta Crystallographica Section A*, 1976, **32**, 751–767.

[27] P. M. Sarte, R. A. Cowley, E. E. Rodriguez, E. Pachoud, D. Le, V. García-Sakai, J. W. Taylor, C. D. Frost, D. Prabhakaran, C. MacEwen, A. Kitada, A. J. Browne, M. Songvilay, Z. Yamani, W. J. L. Buyers, J. P. Attfield and C. Stock, *Physical Review B*, 2018, **98**, 024415.

[28] R. M. Hazen, *American Mineralogist*, 1976, **61**, 266–271.
